# Gene regulatory network inference using fused LASSO on multiple data sets

**DOI:** 10.1038/srep20533

**Published:** 2016-02-11

**Authors:** Nooshin Omranian, Jeanne M. O. Eloundou-Mbebi, Bernd Mueller-Roeber, Zoran Nikoloski

**Affiliations:** 1 Systems Biology and Mathematical Modelling Group, Max Planck Institute for Molecular Plant Physiology, Am Muehlenberg 1, 14476 Potsdam, Germany; 2 Department of Molecular Biology, University of Potsdam, Karl-Liebknecht-Str. 24-25, Haus 20, 14476 Potsdam, Germany

## Abstract

Devising computational methods to accurately reconstruct gene regulatory networks given gene expression data is key to systems biology applications. Here we propose a method for reconstructing gene regulatory networks by simultaneous consideration of data sets from different perturbation experiments and corresponding controls. The method imposes three biologically meaningful constraints: (1) expression levels of each gene should be explained by the expression levels of a small number of transcription factor coding genes, (2) networks inferred from different data sets should be similar with respect to the type and number of regulatory interactions, and (3) relationships between genes which exhibit similar differential behavior over the considered perturbations should be favored. We demonstrate that these constraints can be transformed in a fused LASSO formulation for the proposed method. The comparative analysis on transcriptomics time-series data from prokaryotic species, *Escherichia coli* and *Mycobacterium tuberculosis*, as well as a eukaryotic species, mouse, demonstrated that the proposed method has the advantages of the most recent approaches for regulatory network inference, while obtaining better performance and assigning higher scores to the true regulatory links. The study indicates that the combination of sparse regression techniques with other biologically meaningful constraints is a promising framework for gene regulatory network reconstructions.

Biological systems on different levels of organization, from organelles and single cells to tissues, organs, and entire organisms, constantly sense the environment and modulate their behavior to ensure optimal performance and fitness^1^,^2^,^3^. The sensing of the environment is accomplished via numerous molecular mechanisms which ultimately result in coordinate activation and suppression of, often multiple, regulatory cascades affecting different and mutually dependent cellular processes. By propagating the perceived signal, the expression levels of genes coding for transcription factors (TFs) are adequately altered, leading to changes in the levels of transcripts encoding enzymatic proteins which affect metabolism and organism’s tasks^4^. Therefore, accurate reconstruction of the complete set of regulatory interactions, forming gene regulatory networks, is one of the key tasks in systems biology^5^.

In biological systems, the gene regulatory interactions are transitory, as they depend on different factors, including: developmental, environmental, as well as internal, given by the genetic make-up of the organism^6^. High-throughput technologies for simultaneous measurement of gene expression have been used to capture the transitory behavior of thousands of genes upon internal and external perturbation in different biological systems, from bacteria and yeast to algae, plants, and animals^7^,^8^,^9^. The gathered gene expression levels reflect the underlying regulatory relationships, and, thus, can readily be used to reconstruct the operational regulatory networks.

With the increasing number of performed time-series experiments methods are needed to extract gene regulatory networks supported by *all* gathered data sets simultaneously. These experiments are over different time domains, with different sampling frequency under various conditions, and conducted in different laboratories, which may affect the success of network reconstruction^10^. In addition, each of these experiments is usually accompanied by a corresponding reference control experiment, whose profiles are used to determine differential gene behaviors^11^,^12^.

Reconstruction of gene regulatory networks is a classical problem in computational systems biology, and various methods based on different sets of assumptions and applicable on data from particular experiments have been proposed, critically assessed, and systematically reviewed^13^,^14^,^15^. In general, inference of gene regulatory networks begins with application of a similarity measure of choice (reviewed in^13^) on the investigated data set, resulting in a square similarity matrix. This similarity matrix can be sparsified by retaining only the values which are statistically significant after multiple hypothesis testing.

The resulting (sparsified) matrix usually includes many indirect relationships which need to be detected and removed to increase the power of the network inference approach. Therefore, a major challenge in inferring gene regulatory networks is the identification of direct (causal) relationships between genes. The classical approach to detect indirect relationships is based on partial correlations, which imposes the control of one gene on the relationship of others. For instance, the approaches of^16^,^17^,^18^ attempt to find the causal interactions with partial correlations of small order. Moreover, in Gaussian Graphical Models (GGM)^19^,^20^,^21^,^22^ two genes are deemed to be directly related if their levels are dependent after removing the effect of all other variables^23^. To this end, extensions based on regularization methods (*L*_1_-norm) have been developed^24^. Other approaches rely on information theoretic-based similarity measures^25^,^26^,^27^; for instance, the algorithm for the reconstruction of accurate cellular networks (ARACNE)^25^ is based on Gaussian kernel estimator to determine mutual information between the expression profiles of genes with a sparsity constraint (to filter out non-significant as well as indirect interactions); or context likelihood of relatedness (CLR) which estimates the likelihood of each mutual information score based on the local network context. In addition, approaches rooted in Bayesian Networks (BN) employ probabilistic graphical models in order to infer causal relationships between genes as random variables^28^,^29^,^30^,^31^. Moreover, the combination of *L*_1_-penalized likelihood approach has been applied to structure learning of causal Gaussian Bayesian networks^32^; however, this approach also cannot be used to predict feedback loops and the type of regulatory interactions. The most recent methods aim at detecting indirect relationships based on (weighted) structural investigations of networks following eigenvalue decomposition and its derivatives^33^,^34^, leading to higher inference power than the classically applied approaches. The latter approaches have been thoroughly evaluated for their dependence on parameters and usage with particular data sets, indicating that claims about their performance warrants further examination on additional test cases^35^.

While these approaches aim at reconstructing bilateral relationships, regression-based methods extract one-to-many relationships between genes based on the corresponding transcriptomics profiles^36^,^37^,^38^. A frequently applied regression-based technique in the analysis of high dimensional data involves regularization, whereby the learned models are sparse. Regularized regression models, including ridge and least absolute shrinkage and selection operator (LASSO)^39^, have been already used to infer gene regulatory networks with promising results^40^,^41^. In addition, LASSO has been successfully used in model learning not only in computational biology, but also signal processing, medical imaging, and economics^42^,^43^,^44^. GENIE3 decomposes the inference of gene regulatory networks into *P* (number of genes) regression problems, and tree-based ensemble methods are used to predict the expression profiles of the transcription factor target genes from the expression profiles of the remaining genes^21^. Recently, Qin *et al*. applied *L*_0_, *L*_1/2_, and *L*_1_-regularization models to infer gene regulatory networks^45^. While the performances of these models were comparable to the other compared approaches, increase in accuracy was observed due to the consideration of ChIP-seq/ChIP-chip data in fixing the initial connections between TFs and targets (i.e., initial values for the regression coefficients).

Finally, modern techniques try to harness the “wisdom of crowd” concept by integrating the predictions from different computational approaches (thoroughly evaluated in the DREAM contest^46^) as well as over multiple data sets in a single network termed consensus gene regulatory network^14^,^47^,^48^. For instance, Hase *et al*. grouped the algorithms by applying Euclidean distance on the confidence scores of the links in the corresponding inferred networks^48^. They showed that integration of diverse algorithms outperform the combination of the individual inference methods. Moreover, Villa-Vialaneix *et al*. introduced a GGM based on two penalties: the first supports the global sparsity, while the second minimizes the differences between the coefficients of networks inferred from different data sets and a consensus network^47^. The consensus network was obtained in three ways: by the average of the estimated coefficients over conditions, *a priori* biological knowledge, and pre-calculated coefficients from application of GGM on the combined data sets.

Despite these advances, the problem of accurate reconstruction of gene regulatory networks is far from fully resolved, especially since many of these methods make assumptions about the underlying dynamics of the networks leading to the particular read-outs employed in the reconstruction. Here, we propose a new approach for reconstructing gene regulatory networks, whereby we control for the sparsity while simultaneously harnessing the evidence from multiple data sources. Integrating multiple data sets from different sets of conditions is rooted in the “wisdom of crowds” concept to predict the consensus gene regulatory network. Furthermore, the novelty of the approach lies in combining the similarity in differential behavior between genes with regularized regression methods. To this end, we demonstrate that the approach can be formulated in a form of a fused LASSO model. Our comparative analysis shows that the proposed method is comparable to the most recent contending alternatives as well as classical methods when applied on time-series expression data obtained from two prokaryotic species, *Escherichia coli* and *Mycobacterium tuberculosis*, as well as one eukaryotic species, mouse (*Mus musculus*), but is superior with respect to the performance and assigns higher scores to the true regulatory links. The comparative study includes not only the network deconvolution algorithm and the global silencing (with specification of the used parameter values and precise steps of execution), but also the methods against which these approaches have been gauged in the recent critique^35^—Gaussian graphical models as well as Bayesian networks, ARACNE, CLR, and GENIE3, in addition to the different regularization-based models: *L*_0_, *L*_1/2_ and *L*_1_ (see Methods). Furthermore, although it has been reported that integrating ChIP-seq/ChIP-chip and gene expression data sets enhances the performance of the regularization-based approaches^45^, the majority of existing computational approaches still rely on gene expression data sets; the usage of data sets from one type facilitates the fair comparison of performance of different approaches. The results of the comparison indicate that the combination of sparse regression techniques with time-resolved differential gene behavior is a powerful approach which can be readily extended to include other biologically meaningful constraints in inferring gene regulatory interactions from time-series data.

## Results

### Formulation of the proposed method

LASSO is a linear regression that penalizes the sum of absolute value of the regression coefficients^49^. It relies on combining the *L*_2_-norm, *i.e.*, the residual sum of squares, with the *L*_1_-norm of the regression coefficients, which amounts to sparsity due to shrinking the coefficients towards zero. Moreover, the proposal for considering fusion in the classical LASSO formulation was intended to address problems with a meaningful order in the considered features (*i.e.*, regressors). In fused LASSO, minimization of the *L*_1_-norm is imposed not only on the (ordered) regression coefficients, like in LASSO, but also on the consecutive differences of regression coefficients based on the assumed order of corresponding regressors^50^.

In this section we formulate the approach which extends the concept of fused LASSO by incorporating information about the similarity of differential behavior for the response and regressor genes. Here, every gene is taken as response and the genes which code for transcription factors (known as TF genes) are used as regressors. The fusion imposes the similarity constraint on the obtained networks from each data set. In this way, the approach ensures sparsity, commonly observed in gene regulatory networks, with biologically meaningful data-driven evidence for the inferred relationships. In addition, we introduce the experimental scenario and type of data on which the approach is readily applicable.

### Experimental scenario and weight matrices

**Figure 1:**
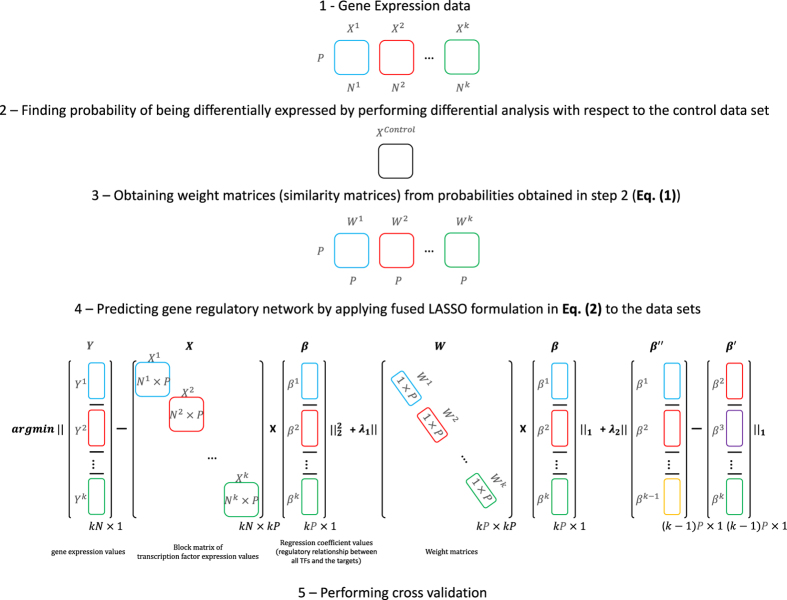
Representation of the analysis flow, data sets and their transformation for usage in the model. Representation of condition and control data sets *X*^*i*^ and *X*^*control*^, respectively, containing the expression levels of *P* genes, as regressors, over *N*^*i*^ time points, 1 ≤ *i* ≤ *k*. Block matrix *X* contains the gene expression values *X*^*i*^, 1 ≤ *i* ≤ *k*, for the regressor genes (transcription factors) in the diagonal. *Y* includes the profiles *Y*^*i*^, 1 ≤ *i* ≤ *k*, for the response genes. Weight matrices *W*^*i*^ contain the similarity of differential behavior between the response gene and each of the regressors based on data sets from *k* perturbation experiments and the corresponding controls. Regression coefficient values *β*^*i*^, 1 ≤ *i* ≤ *k*, consists of regulatory relationship between a TF and its targets from *k* perturbation experiments.

For consistency throughout the text, all superscripts refer to the experiments/conditions and subscripts denote genes. We will define the approach for *P* genes used as regressors, and a single gene used as a response. The approach assumes that *k* gene expression data sets, denoted by *X*^*i*^, are gathered under *k* different conditions alongside corresponding reference (*control*) data sets *X*^*c*,*i*^, 1 ≤ *i* ≤ *k*, all of them containing the expression levels of *P* genes over *N*^*i*^, 1 ≤ *i* ≤ *k*, time-points or perturbations (Fig. 1, step 1 and 2). Note that the data sets from the *k* different conditions do not have to be over the same time domain or with the same sampling frequency; the only requirement is that each data set allows to reliably obtain the corresponding covariance matrix for the *P* genes. In addition, the profiles *Y*^*i*^ for the single response gene over the *k* conditions together with the corresponding control profiles *Y*^*c*,*i*^, 1 ≤ *i* ≤ *k* are given.

Since reference data sets *X*^*c*,*i*^, 1 ≤ *i* ≤ *k*, are available, we first determine the differential behavior for each of the regressors as well as for the response in each condition. To this end, we rely on the gene-specific B-statistic^11^ corresponding to the log-odds that the gene is differentially expressed at a given time point of a particular condition in comparison to the control. Let 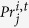, 1 ≤ *j* ≤ *P*, 1 ≤ *i* ≤ *k*, 1 ≤ *t* ≤ *N*^*i*^ be the probability that gene *j* at time point *t* under condition *i* is differentially expressed. Then the matrix *Pr*^*i*^ of dimension 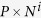 gathers the probabilities for the time-dependent differential behavior of the considered genes, estimated by the corresponding B-statistics. The B-statistics was estimated for each gene and at each time point by comparing data set *X*^*i*^ from the treatment and the respective control *X*^*c*,*i*^, 1 ≤ *i* ≤ *k*.

The derived probabilities can be used to define weight matrices for each condition *i* (1 ≤ *i* ≤ *k*), denoted by 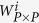, which capture the information on the similarities between the response gene and each of the regressor genes based on their differential behavior (Fig. 1, step 3), by using the following:


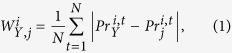


where 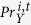 and 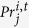 are the probabilities of differential behavior for the response gene *Y* and the regressor gene *j* (1 ≤ *j* ≤ *P*), respectively, at time point/perturbation *t*.

If the value of 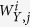 is close to zero, the *j*^*th*^ regressor gene has, on average, a similar differential behavior to the response over all time points/perturbations in a data set from one condition. More specifically, 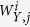 is close to zero if either both genes are differentially expressed or they are both not affected by the perturbation. Due to the symmetric nature of the absolute value in Eq. (1), the weight matrix *W*^*i*^ is symmetric with zeros along the diagonal.

### Regression-based model

Having *k* time-resolved gene expression data sets including *P* + 1 genes and *N*^*i*^, 1 ≤ *i* ≤ *k*, time points together with the corresponding control data set(s), we aim at formulating a model which captures the following three criteria: (1) the expression levels of each gene should be explained by the expression levels of a small number of transcription factor (TF) coding genes, *i.e.*, the corresponding regression should be sparse to reduce the number of false positives and likely increase the detection of direct relationships; (2) the regulatory networks should be inferred simultaneously over the given *k* data sets to simultaneously explain all analyzed conditions; and (3) a direct link should be preferred for genes which exhibit similar differential behavior over the considered conditions, since a differentially behaving gene (TF) is likely to alter the behavior of a direct target. Here, for the purpose of building the regression models, we focus on using the data sets from the *k* conditions only, as they are potentially more informative about the responsive genes in comparison to the reference state. In addition, the control data set enter the modeling through the probabilities of differential behavior, and are, thus, also employed in the reconstruction of the gene regulatory network.

The first criterion can be captured by the classical LASSO, so that the regressors with non-zero regression coefficients are considered to be involved in a direct relationship with the response gene, yielding the gene regulatory network. To address the second criterion of simultaneously inferring *k* networks from the *k* different data sets, one has to ensure that: (*i*) the integration and transformation of the data should be performed in such a way that the LASSO penalty is simultaneously imposed to all data sets, and (*ii*) the *k* reconstructed networks should be as close as possible in terms of edges (*i.e.*, relationships) their strength given by the coefficients, and the sign of the relationship (activating or repressing).

The integration and transformation of the data sets is achieved as follows: the *k* transcriptomics data sets are combined into a single block matrix *X* of dimensions *kN* × *kP*, where 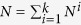, *Y* is a collection of the profiles for the response gene from the different conditions, resulting in a vector of dimension *kN* × 1. As a result, the estimated coefficients form a *kP* × 1-vector corresponding to the edges between the response gene *Y* and regressor genes in the *k* network. The vector of estimated regression coefficients is represented with *β*.

To ensure that the *k* reconstructed networks are as close as possible, we add the fusion LASSO term 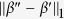, where *β*′ = [*β*^1^, *β*^2^, …, *β*^*k* − 1^]^*T*^, *β*′ = [*β*^2^, *β*^3^, …, *β*^*k*^]^*T*^ and the order of the *k* data sets is arbitrarily selected. In a regression setting, this fusion term imposes the constraint that the sum of absolute differences between the estimated coefficients of the same regressor over the consecutive data sets, with the assumed arbitrary order, is minimized (Supplementary Fig. S3 and Supplementary Table S8). This idea differs from the most recent approach whereby networks reconstructed from different data sets are individually obtained and later combined in a consensus network by existing techniques^14^,^51^.

The third criterion about the similarity of differential behavior between the response and regressors implies the inclusion of the weight matrix *W*^*i*^, 1 ≤ *i* ≤ *k*, so that the regressor of higher explanatory power, which are associated with non-zero regression coefficients, over the multiple data sets are less penalized in the fused LASSO regression. This can be achieved by multiplying the regression coefficients with the weight matrix *W* (of size *kP* × *kP*), gathering the similarity of differential behavior between each of the *P* regressors and the single response gene under the *k* conditions. As a result, the expression 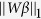 is included as a modified penalty in the regression.

Therefore, the final model for reconstructing the gene regulatory interactions is given by the following fusion LASSO formulation over the *k* given data sets (Fig. 1, step 4):


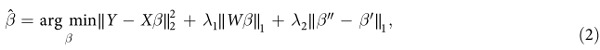


where *Y* is the response gene which is regulated by the regressors with non-zero regression coefficients.

### Gene expression data sets

#### Escherichia coli

The responses of *Escherichia coli* to stress conditions have already been well-investigated, resulting in characterization of the general and condition-specific components that regulate transcriptional changes underlying the adjustment to changing environments^52^,^53^. The gathered data sets provide an excellent test case to which the performance of the proposed method and the contending alternatives can be readily compared.

The time-resolved transcriptomics data sets gathered with microarray technology were obtained from^54^, where changes in the expression of genes from *E. coli* strain MG1655 were monitored under four stress conditions, including: non-lethal temperature shifts, *i.e.*, heat and cold treatment, oxidative stress (by adding hydrogen peroxide), lactose-diauxic shift (*i.e.*, change of primary carbon source) relative to cultures grown under optimal conditions, referred to as control. All cultures were grown simultaneously in the same condition and different perturbations were applied at the early mid-log phase (OD 0.6). The sampling was carried out from time points 10–50 min post-perturbation (at 10 min intervals) and two control time points before each perturbation for all considered conditions (data are available at http://www.ncbi.nlm.nih.gov/geo/query/acc.cgi?acc=GSE20305).

The reasons for selecting and using these data sets are that the predictions can be readily validated against the gold standard benchmark for *E. coli* provided by the DREAM5 challenge^14^ as well as the experimentally verified regulatory network interactions from RegulonDB^53^. In RegulonDB, the information about the effect of the regulators (in terms of activating and repressing interactions) on the target genes is also provided. Moreover, these data sets satisfy the requirements of the proposed approach, since the single control can be used to establish the differential gene behavior across the data sets upon application of the stresses. In addition, the data are gathered from the same laboratory following the same protocol, thus, reducing the level of noise. Finally, and most importantly, we aimed at using real-world data sets to estimate the actual performance of the proposed method in a realistic setting rather than from simulated instances.

#### Mycobacterium tuberculosis

*Mycobacterium tuberculosis* (*MTB*) is a pathogenic bacterium whose gene regulatory network is poorly understood. However, recently Galagan *et al*.^55^ made the first step in reconstructing the complete regulatory network of *MTB* based on the ChIP-seq and microarray gene expression data gathered under conditions of hypoxia and re-aeration. They performed time-resolved transcriptomics experiments in which the expression level of genes were measured at 1, 2, 3, 5, and 7 days after culturing *MTB* strain H37Rv in bacteriostatic hypoxia conditions. The samples were then placed back in the aerobic rolling culture and the gene expression levels were measured after 1, 2, 4, 5, and 7 days of re-aeration. The expression levels were also measured at time point 0 which is considered as the control data set for the differential analysis (data are available at http://www.ncbi.nlm.nih.gov/geo/query/acc.cgi?acc=GSE43466).

**Figure 2:**
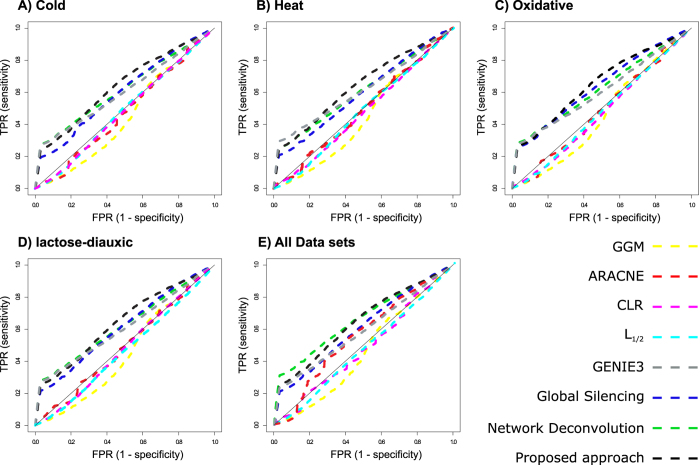
ROC curves for the methods considered in the comparative analysis (*E. coli* data sets). ROC curves are shown for the gene regulatory networks predicted based on the data sets from (**A**) cold, (**B**) heat, and (**C**) oxidative stress as well as (**D**) lactose-diauxic shift in addition to the (**E**) combination of all four data sets by using the following methods: GGM, ARACNE, CLR, *L*_1/2_, GENIE3, Global silencing, Network deconvolution, and the proposed approach based on the fused LASSO which simultaneously considers all four data sets. Due to the high similarity in the performance of the regularization-based models, the ROC curves of *L*_1/2_ model is illustrated as the representative to avoid curve overlapping. The color of the dashed lines represents the methods. TPR and FPR stand for true positive rate and false positive rate, respectively.

The reasons for selecting and using these data sets are that: (i) the predictions can be validated with respect to recent real-world data sets from non-model species, (ii) it satisfies the requirements of our approach for inferring the gene regulatory network from multiple data sets, as the gathered time series can be considered as two separate time-resolved data sets (conditions): hypoxia and re-aeration while including time point 0 as a control, and (iii) the predictions can be readily compared to the small sub-network of *MTB* which was partially verified through experiments in^55^ (Fig. 2 in^55^).

#### Mus musculus

*Mus musculus* represents a model organism for understanding human biology and disease; therefore, studying its large-scale gene regulatory network is an important challenging task. Many experiments have been performed on mouse embryonic stem (ES) cells to explore the details of their pluripotency and ability to self-propagate and renew. To this end, Sene *et al*.^56^, measured the expression level of genes from three genetically distinct mouse ES cell lines (R1, J1, and V6.5) during differentiation at 11 time points: 0 h, 6 h, 12 h, 18 h, 24 h, 36 h, 48 h, 4 d, 7 d, 9 d, and 14 d. The expression levels at time point 0 are considered as the control data set for the differential analysis (the processed data has been downloaded from http://www.maayanlab.net/ESCAPE/browse.php).

This data set provides a good test case for comparing the performance of the compared approaches for gene regulatory network extraction in a higher organism. Moreover, the literature-based embryonic stem cell network is used as a global standard network to validate the predictions obtained from the compared approaches (downloaded from iScMiD (Integrated Stem Cell Molecular Interactions Database) http://amp.pharm.mssm.edu/iscmid/literature/index.htm).

### Comparative analysis

For the comparative analysis we considered the most recent as well as the state-of-the-art approaches which include two criteria, sparsity of the network and removal of indirect relationships. To this end, we used the following approaches: the global silencing^33^, network deconvolution^34^, Gaussian Graphical Models (GGM)^19^, mutual information (ARACNE^25^, and CLR^26^), Bayesian Network (catnet)^57^, the GENIE3 approach^21^, and different regularization-based models^45^ (more details in the Methods section).

#### Escherichia coli

The resulting networks from these approaches were compared to the network including experimentally verified regulatory relationships from RegulonDB and the gold standards from the DREAM5 challenge. The findings were summarized in terms of the resulting true positive and false positive rates, which resulted in the corresponding ROC (receiver operating characteristic) curves illustrated in Fig. 2 (based on the used threshold value for retaining a weighted edge in the network). We used the R package minet^58^ to plot the ROC curves, and the statistics obtained by using this package are summarized in Table 1. As illustrated in Fig. 2, the proposed approach allowed the prediction of regulatory relationships at a higher percentage of true positives in comparison to the GENIE3, network deconvolution and the global silencing methods, while GGM, ARACNE, CLR, and regularization-based models performed poorly for accurate inference of the regulatory network. Moreover, it is evident that, on the used data sets, the proposed method based on the extension of the fused LASSO performed similarly to the considered contending approaches when only the connectivity (*i.e.*, presence of an edge/relationship) is considered. To further quantify the performance of the compared methods, we calculated the area under the ROC curves (AUROCs), the area under PR (precision-recall) curves (AUPRs), and the true positive rates (TPR) at low false positive rate (most of the compared methods showed maximum difference between TPR and FPR at FPR = 0.03), presented in Table 1. We used the R package pROC^59^ to estimate the AUROC curves and their respective confidence intervals (CI) (Supplementary Table S1). The other statistics resulting from the comparison of the considered approaches are summarized in Supplementary Table S1. Altogether, our findings demonstrate that the performance of the proposed approach is statistically similar to the performance of GENIE3 and network deconvolution, while outperforming the remaining of contending methods.

In our approach, the network over all data sets was obtained by taking the maximum or the average of the corresponding regression coefficients (see Supplementary Fig. S1 (a) for illustration). Since the distribution of the differences between the regression coefficients obtained based on each of the four data sets for the same response and regressors are almost identical (see Supplementary Fig. S1 (b)), the two networks are not expected to differ. Indeed, the comparison between the two networks obtained by taking the average and the maximum of the regression coefficients does not show a significant difference with respect to the AUROC values (*p*-value = 0.6257, see Supplementary Table S1).

The performance of each method over the four data sets and their combination was summarized by the geometric mean for the five corresponding AUROCs and AUPRs, while the OverallScore is the average of the calculated AUROCscores and AUPRscores^14^:


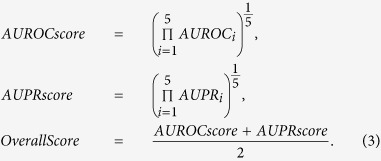


The OverallScore in Eq. (3) can be used to rank the compared methods, such that a larger value corresponds to a better-performing method. As shown in Table 1, the performance of our method was superior to that of other contending methods based on the OverallScore. Moreover, the standard deviation of the AUROC curves for the proposed method is smaller in comparison to the contenders (see Supplementary Table S1). The stability of the obtained networks further supports the usage of the fusion term in the proposed approach, which was considered for the very purpose of producing similar networks across different data sets.

We next determined the smallest normalized weight at which the first true positive edge can be detected—termed *selector value*; selector values closer to 1 indicate that the true positive edges are likely assigned higher weights. As shown in Table 2, the proposed method performed the best for networks obtained over all data sets. Moreover, our approach is in the two best performing methods when considering individual data sets. The discrepancy can be explained by the inclusion of the fusion term which insists on context-independent regulatory relationships. Since the edge weights of the networks obtained by ARACNE with each of the four data sets were identical, the corresponding selector values are not informative and cannot be used in the comparison. Interestingly, the network deconvolution and the global silencing did not perform well with respect to the selector value, despite recent claims on synthetic data. The reasonably high *selector values* obtained from regularization-based models further confirm the power of regularization-based approaches at assigning higher scores to the true positive edges.

Furthermore, the accuracy of inferred gene regulatory networks depends on the ability to predict the type of the regulatory relationships—activating or repressing. Therefore, we compared the performance of the methods regarding the type of regulatory relationships predicted. To this end, we used the network from RegulonDB which includes in total 4566 relationships. Table 3 summarizes the percentages of the predicted true activating and repressing regulatory interactions for the four data sets and the considered methods. In addition, it includes the percentages of the true positive and true negative relationships predicted by different methods, irrespective of the type of regulation.

ARACNE and CLR as well as GENIE3 are not able to infer the type of regulatory relationships. As mentioned in Section 3.2, the original algorithms for the global silencing and the network deconvolution do not provide the sign of interactions, since they aim at ranking the regulatory interactions. As shown in Table 3, our findings indicate that the performance of the proposed method with respect to the prediction of the type of regulatory relationships is promising. In addition, GGM as well as regularization-based models resulted in the largest fraction of true negative edges as well as the smallest fraction of true positive edges. The deconvolution method, however, outperformed the rest of the approaches with respect to the fraction of true positive edges for cold and oxidative stress as well as lactose-diauxic shift (albeit with much smaller selector values, see Table 2).

**Figure 3:**
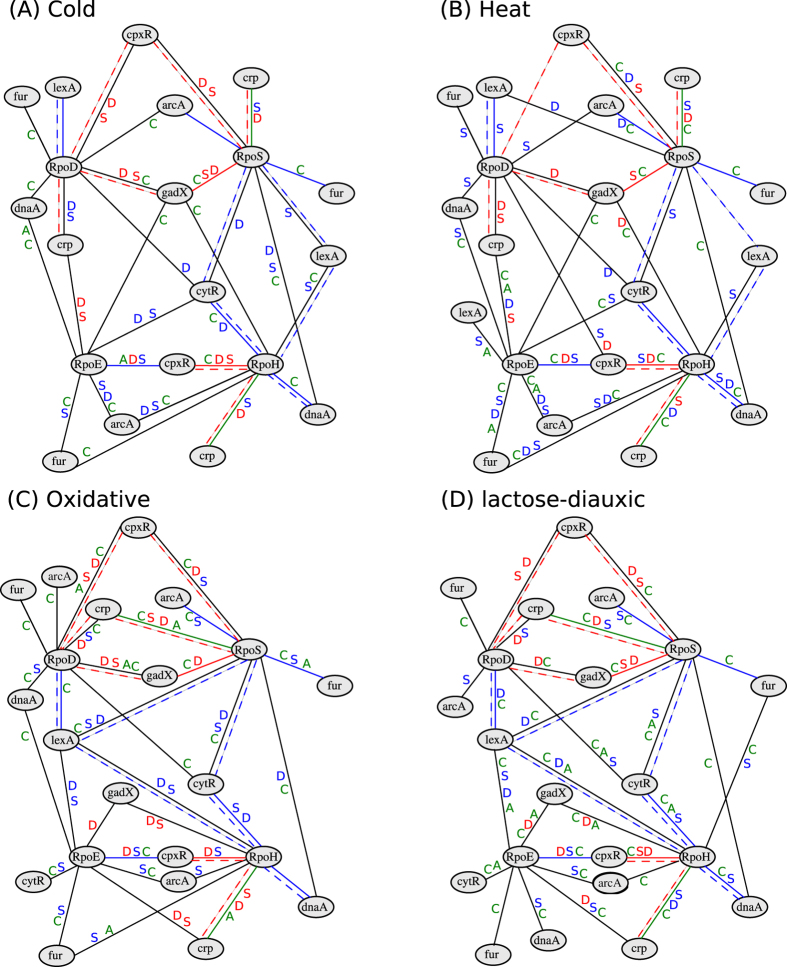
Sub-networks including sigma factors. The gene regulatory network for four sigma factors, RopD, RopE, RopH, and RopS, together with their experimentally verified interactions obtained from RegulonDB^53^. The colored edges belong to the sub-network retrieved from RegulonDB, where red edges denote activating, while blue edges indicate repressing regulatory relationships. The edges marked in green are of unspecified regulatory type. If an edge was predicted by a method but is not included in the network from RegulonDB, it is colored in black. The predicted edges for ARANCE, catnet, the network deconvolution and global silencing methods are marked by ‘A’, ‘C’, ‘D’ and ‘S’, respectively, next to the corresponding edges. The letters are color-coded—red, blue or green fonts represent activating, repressing or unspecified relationships, respectively. The dotted edges denote the relationships predicted by the proposed approach. Illustrated are the predicted regulatory relationships and their types based on data from (**A**) cold, (**B**) heat, (**C**) oxidative stress, and (**D**) lactose-diauxic shift time-series experiments.

In addition, to specifically inspect the efficiency of the inferred networks, we selected a subnetwork from RegulonDB including four sigma factors, RopD, RopE, RopH, and RopS, included in the analyzed data sets. Sigma factors are proteins required for initiation of RNA synthesis^60^, and their activities depend on the environmental conditions. While RopD is the primary sigma factor which transcribes most of the genes, the activities of the other three are environment-specific; for instance, RopE is the extreme heat stress sigma factor, RopH is the heat stress sigma factor activated upon heat exposure, and RopS is the stationary phase sigma factor. Interestingly, for the four sigma factors, no edges were predicted by GGM. With respect to the six other approaches as well as catnet (applied only on genes included in the illustrated networks), by inspecting Fig. 3 (for the sake of readability, the resulting networks for CLR, GENIE3 , 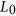, 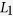, and 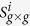-regularization models are shown in the Supplementary Fig. S2), it becomes evident that the proposed approach predicted a considerably smaller number of false positives, due to the sparsity constraints, and a comparable number of true positives, due to the regression-based formulation. In addition, as a result of the imposed constraints in the fused LASSO formulation, the networks extracted for the four different data sets based on our method are exactly the same (except for slight differences in the weights - see Supplementary Fig. S1), which is biologically expected. However, this was not the case for the networks reconstructed by ARACNE, CLR, GENIE3, catnet, the network deconvolution and the global silencing methods; in addition, the networks from GENIE3, catnet and the deconvolution method were the densest over all data sets. Altogether, regularization-based models largely underestimated the true positive edges, although they were consistent in predicting the edges for each single data set. The proposed approach performed particularly well with respect to predicting the type and not only the presence of a regulatory relationship, evident in the cases of the RpoH and RpoD sigma factors.

Finally, we obtained the run time of each inference method which is presented in Supplementary Table S2. Since the proposed method includes many independent regression models, we parallelized the approach (as described in Methods). Therefore, the run time of the proposed method is only provided for performing a single regression. Clearly, the algorithms which rely on matrix inversions in the presence of hidden parameters (e.g., network deconvolution) are faster in comparison to the proposed method which requires solving multiple regressions. While cross validation is expected to increase the computational demand, if a single value is used across all genes (*i.e.*, models) the regulatory interaction in the vicinity of a gene can be inferred in a drastically reduced time in order of a few seconds.

#### Mycobacterium tuberculosis

Motivated by the predictions from applying the proposed approach on the combination of all *E. coli* data sets, we next investigated the comparative analysis only on the combination of both data sets from *MTB* (*i.e.*, hypoxia and re-aeration). To obtain gene regulatory networks, we applied all compared methods to the pre-processed gene expression values as well as the log 2-transformed gene expression fold changes between control (time point 0) and the hypoxia and re-aeration time-series samples. The ROC analysis for the compared methods was then obtained by using the R package minet^58^ for both: the top 31 (as the gold standard network includes 31 edges) and 100 highly ranked edges, and the corresponding statistics are summarized in Supplementary Table S3. It is evident that the proposed approach allowed the prediction of regulatory relationships at a higher percentage of true positives in both cases of using gene expression values and log 2-transformed gene expression fold changes when comparing to the performance of GENIE3, network deconvolution and global silencing methods. Likewise with *E. coli*, GGM, ARACNE, CLR, and regularization-based models performed poorly for accurate inference of the regulatory network; however, the performance of the majority of the compared methods was increased upon applying to the log 2-transformed gene expression fold changes. In addition, the selector values for all compared methods were the same and equal to one.

Furthermore, to specifically inspect the efficiency of the inferred networks, we investigated on three experimentally verified TF regulatory interactions that are highlighted in the Result section from Galagan *et al*. (page 180,^55^): TF Rv0081 negatively regulates TFs Rv3597c and Rv3416 (whiB3) (Rv0081 → Rv3597c (Lsr2), Rv0081 → Rv3416 (whiB3)), and TF Rv3133c and Rv2034 (DosR) negatively regulate each other (Rv3133c ↔ Rv2034 (DosR)). All interactions and their regulatory types were successfully predicted by the proposed approach with sufficiently high ranks (Supplementary Table S4). Global silencing as well as network deconvolution methods were not successful with respect to the type of regulatory interactions and obtained rather low ranks, while GENIE3 predicted all interactions with high ranks but is not able to infer the type of regulatory relationships. Likewise, GGM, ARACNE, and CLR were not successful in predicting the interactions, while regularization-based models performed very inconsistent with respect to the sign and predicting true edges.

Moreover, we counted the number of predicted true interactions in the top 100 highly ranked edges (Supplementary Tables S4, S5, and S6) considering the gold standard sub-network which includes 31 interactions (Fig. 2 in^55^). It is evident that the proposed approach obtained the highest overlap with the gold standard while the minimum rank of the intersected interactions is remarkably high (above 0.6). Finally, the networks predicted by the compared approaches were filtered by the corresponding median edge ranks and the ROC analysis has been performed to the resulting sub-networks. Here, too, our proposed approach resulted in the highest AUROC and AUPR values while the edges in the filtered sub-network have considerably higher ranks (above 0.6075 and 0.379 when applied to gene expression levels and log 2-transformed gene expression fold changes, respectively) in comparison to the median ranks of the other contending methods.

#### Mus musculus

Finally, to verify the performance of the proposed approach in a higher organism, we performed a comparative analysis on the combination of tissue-specific time-series data sets from three genetically distinct mouse ES cell lines during differentiation. We applied all compared methods to the log 2-transformed gene expression fold changes between control (time point 0) and the rest of time-series from the corresponding ES cell lines, motivated by the resulting improvement in the performance of the inference approaches applied to the *MTB* data sets. The ROC analysis for the compared methods was then carried out on the top 248 (as the gold standard network includes 248 edges) and 500 highly ranked edges, and the corresponding statistics are summarized in Supplementary Table S7.

It is evident that the proposed approach allowed the prediction of regulatory relationships at a higher percentage of true positives when compared to the performance of GENIE3, network deconvolution and global silencing methods. Likewise with other data sets CLR as well as regularization models performed poorly for accurate inference of the regulatory network; however, the performance of the GGM is increased in comparison to its performance on the other data sets, while ARACNE failed to predict any true positive edge. In addition, the selector values for most of the compared methods were high and close (or equal) to one.

Moreover, we counted the number of predicted true interactions in the top 248 highly ranked edges (Supplementary Tables S7 and S9) considering the gold standard sub-network (which includes 248 interactions). It is evident that the proposed approach obtained the highest overlap with the gold standard, while the minimum rank of the intersected interactions is remarkably high (above 0.5). Finally, the networks predicted by the compared approaches were filtered by the corresponding median edge ranks and the ROC analysis has been performed to the resulting sub-networks. Once again the proposed approach resulted in the highest AUROC and AUPR values, while the edges in the filtered sub-network have similar ranks as the other predicted sub-networks by the other contending methods.

## Discussion

Here we proposed a novel computational method for the reconstruction of gene regulatory networks which harnesses the information available in multiple data sets (time-series or perturbations) from different conditions. The approach is based on a fused LASSO formulation which incorporates not only the constraint that the sparse networks extracted from the different data sets are supposed to be as similar as possible, but also the requirement that the genes predicted to participate in a regulatory interaction should exhibit similar differential behavior under different conditions. We applied the proposed method to the different real-world gene expression data sets from two bacteria as well as *Mus musculus*. The thorough comparative analysis with the most recent as well as the state-of-the-art approaches demonstrated that the proposed method (i) showed highest performance on individual data sets (*E. coli* data set), (ii) outperformed the other contending approaches with respect to the predicted networks from MTB as well as *Mus musculus* on combination of all data sets, (iii) was promising for inferring the type of regulatory interactions (*E. coli* data set), (iv) partially outperformed the alternatives on the combined data sets (*E. coli* data set), (v) was superior for selector values, and finally (vi) was more stable with respect to the inferred networks from single data sets. In addition, solving the problems relying on computing inverse matrices (e.g., inverse of covariance, used in GGM and global silencing) or distance matrices (e.g., based on mutual information) can be solved in shorter time than individual regressions for each gene (as a response in the regression model), but since covariances for most of the gene expression data sets are ill-conditioned, the proposed approach opts for regularized regression. In addition, with respect to the results from *MTB*, we could show that the performance of the majority of the inference approaches increased by using fold changes (obtained from differential analysis) rather than gene expression values; however, this needs to be further investigated and specifically evaluated. Finally, and of most interest, the proposed approach provides predictions of regulatory interactions which can be readily interpreted due to the incorporation of biologically meaningful constraints in the usage of data.

## Methods

### Data preprocessing

#### Escherichia coli

We used the preprocessed and normalized data for each data set (detailed description in^54^). The final data sets contain the expression of 4400 genes at the aforementioned time points. To test our approach, we used the set of transcription factors present in RegulonDB (TF-gene and TF-TF interactions) as well as DREAM5 challenge. Based on the available microarray data sets, the profiles of altogether 210 regulators (TFs) and 1561 non-regulators were considered for the inference of the gene regulatory networks.

The model proposed in Eq. (2) was then solved for each of the 1561 genes as responses in the regression and the 210 TFs as regressors. We calculated the weight matrices *W*^*i*^, 1 ≤ *i* ≤ 4, and the transformations were applied on the four data sets. The weight matrices were calculated using the limma package^61^ in R^62^. The probabilities of being differentially expressed at each time point were estimated by the B-statistic of a contrast (for a time point).

#### Mycobacterium tuberculosis

We used the preprocessed and normalized data for each data set (available in ArrayExpress with the accession number E-GEOD-43466, http://www.ebi.ac.uk/arrayexpress/experiments/E-GEOD-43466/). The final data sets used for inferring gene regulatory network include only 20 TFs present in the experimentally verified TF regulatory sub-network available in^55^ (Fig. 2, page 180).

The model proposed in Eq. (2) was then solved for each of the 20 TFs considered similarly as responses and regressors in the regression. We calculated the weight matrices *W*^*i*^, 1 ≤ *i* ≤ 2, and the transformations were applied on the two data sets. The weight matrices were calculated in the same fashion as for *E. coli*.

#### Mus musculus

We used the preprocessed and normalized data for each data set (available in ESCAPE databse, http://www.maayanlab.net/ESCAPE/browse.php). We used the gene expression data obtained from MOE430A array due to measuring genes which are better characterized. The final data sets contain the expression of 8274 genes (the genes with more than one expression profiles in the array were removed) at the aforementioned time points. To test our approach, we used the set of transcription factors present in the literature-based ES network (iScMiD, http://amp.pharm.mssm.edu/iscmid/literature/index.htm). Based on the available microarray data sets, the profiles of altogether 26 regulators (TFs) and 61 genes were considered for the inference of the gene regulatory networks.

The model proposed in Eq. (2) was then solved for each of the 61 genes as responses in the regression and the 26 TFs as regressors. We calculated the weight matrices *W*^*i*^, 1 ≤ *i* ≤ 3, and the transformations were applied on the three data sets. The weight matrices were calculated in the same fashion as for *E. coli*.

### Methods used in the comparative analysis

#### Escherichia coli

For each of these methods used in the comparative analysis (except catnet, due to its time complexity), we employed the data sets from each perturbation separately and all together, and divided the profiles into the 210 TFs (regulators) and 1561 non-regulator genes. For the network deconvolution and the global silencing methods, we proceeded as follows: First, for the given data set, we calculated the Pearson correlation coefficients matrix 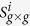
63. Given *g*_1_ regulators and *g*_2_ non-regulators, with *g* = *g*_1_ + *g*_2_, the correlation matrix can be modified as 
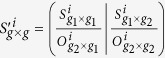
, where *O* denotes the zero matrix, to include biological roles (TF and non-TF genes). We then applied the network deconvolution and global silencing methods to the modified correlation matrix *S*′^*i*^, 1 ≤ *i* ≤ 4 from the four data sets (*i.e.*, cold, heat, oxidative stress, and lactose-diauxic shift) and the combination of all, resulting in five gene regulatory networks per method. However, the global silencing depends on finding the inverse of the correlation matrix which is rank-deficient in the case *p* >> *n*, like with the data analyzed here. Since finding an inverse for a rank-deficient matrix is an ill-posed problem, we resolved it by adding a noise term which renders the matrix positive-definite. In the comparative analysis, we selected the best result from 10 runs of the procedure as a final outcome used in the comparative analysis.

We used the package GeneNet^64^ to infer gene regulatory networks based on the GGM proposed in^19^ to also obtain four gene regulatory networks for each of the stresses and one network for the combination of data sets. Likewise, R packages minet^58^ and catnet^57^ were applied to reconstruct gene regulatory networks based on ARACNE^25^ (as well as CLR^26^) and categorical Bayesian network^65^, respectively. For GENIE3, we used the corresponding online tool available in GP-DREAM^14^,^66^ (http://dream.broadinstitute.org/gp/pages/index.jsf). Finally, for different regularization models; *L*_1_, *L*_1/2_, and *L*_0_, we used the available source code from http://jjwanglab.org/LpRGNI/.

To be consistent with the obtained edge weights from each network inference method, we rescaled them to positive values between 0 and 1 (by dividing the absolute value of the edge weights by the largest absolute value of the edge weights). Then the edges were sorted in a decreasing order of their weights, and the top 100,000 highly ranked edges were selected for the rest of the statistical assessments. We note that the resulting networks from global silencing and network deconvolution are rescaled by default. In addition, the actual edge weights (before rescaling) were stored for each network to estimate the percentages of predicted true positive and negative regulatory effects (*i.e.*, activation and inhibition, respectively). The inference of regulatory type, in terms of activation or inhibition, would not be possible without capturing the edge weights before rescaling, described above.

#### Mycobacterium tuberculosis

The comparative analysis was performed only on the combination of all data sets resulting in one gene regulatory network per method. As the gold standard network for *MTB* includes only transcription factors, we applied the network deconvolution and the global silencing methods to the correlation matrix *S*′^*i*^, 1 ≤ *i* ≤ 2 from the combination of both data sets (*i.e.*, hypoxia and re-aeration) without further modification. The rest of the analysis was performed in the same way as for *E. coli*, except the selection of highly ranked edges for which due to the smaller network size, we selected the top 31 (the size of gold standard network) and 100 highly ranked edges.

#### Mus musculus

The comparative analysis was performed only on the combination of all data sets resulting in one gene regulatory network per method. The inference of the gene regulatory networks and the comparative analysis of the compared approaches were performed in the same manner as for *E. coli*, except the selection of highly ranked edges for which due to the smaller network size, we selected the top 248 (the size of gold standard network) and 500 highly ranked edges.

### Implementation notes for the proposed approach

To implement the modified fused LASSO approach for reconstruction of gene regulatory networks over different data sets, we defined a penalty matrix *D* composed of two blocks: The first block *D*_1_ is a diagonal *kP* × *kP* matrix for the LASSO penalty which includes *W* in diagonal, while the second block *D*_2_ corresponds to the fusion penalty, expressed as:


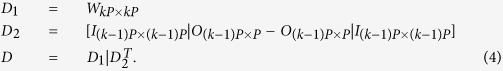


To solve the proposed extension to the fused LASSO, we used the lqa package in R^67^, with remarkable computational performance due to the Cholesky decomposition^68^ which requires 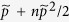 operations, in which *n* is the number of observations and 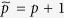 while *p* is the number of regressors. We also slightly changed the function fused.lasso in lqa package to allow inclusion of the penalty matrices defined in Eq.(4). The regression coefficients were robustly estimated by 10-fold cross validation based on the optimum values for *λ*_1_ and *λ*_2_ from the sets {0.05, 0.1, 0.5, 1, 1.5} and {0.1, 0.5, 1, 1.5, 2}, respectively. To further speed up the algorithm, we used mclapply function from R package parallel^62^. In addition, we used minet^20^ and pROC^59^ packages in R to draw ROC curves and estimate statistics for them.

The code snippets for all approaches used in this study is available at http://mathbiol.mpimp-golm.mpg.de/Mul-fLASSO/index.html. The complete implementation of the proposed approach for the data sets used in this study as well as the general analysis flow for applying the proposed approach on new data sets are available at https://github.com/omranian/inference-of-GRN-using-Fused-LASSO.

## Additional Information

**How to cite this article**: Omranian, N. *et al*. Gene regulatory network inference using fused LASSO on multiple data sets. *Sci. Rep.* **6**, 20533; doi: 10.1038/srep20533 (2016).

## Supplementary Material

Supplementary Information

## Figures and Tables

**Table 1:** ROC-based statistics for the compared methods (*E. coli* data sets).

Methods	Cold			Heat			Oxidative			Lactose			All data sets				
	AUROC	AUPR	TPR (FPR = 0.03)	AUROC	AUPR	TPR (FPR = 0.03)	AUROC	AUPR	TPR (FPR = 0.03)	AUROC	AUPR	TPR (FPR = 0.03)	AUROC	AUPR	TPR (FPR = 0.03)	OverallScore	sd (AUROC)
GGM	0.451	0.0012	0.016	0.450	0.0012	0.013	0.458	0.0012	0.015	0.454	0.0012	0.015	0.460	0.0012	0.015	0.228	0.004
ARACNE	0.478	0.0025	0.023	0.497	0.0027	0.05	0.486	0.0026	0.032	0.495	0.0027	0.025	0.558	0.0031	0.010	0.252	0.031
CLR	0.486	0.0013	0.028	0.477	0.0014	0.018	0.472	0.0013	0.033	0.483	0.0013	0.027	0.483	0.0026	0.046	0.241	0.005
*L*_1_	0.487	0.0013	0.015	0.487	0.0013	0.015	0.479	0.0013	0.015	0.469	0.0013	0.017	0.479	0.0013	0.018	0.240	0.007
*L*_1/2_	0.487	0.0013	0.015	0.487	0.0013	0.015	0.478	0.0013	0.014	0.467	0.0013	0.017	0.473	0.0013	0.015	0.240	0.009
*L*_0_	0.487	0.0013	0.015	0.487	0.0013	0.015	0.478	0.0013	0.014	0.467	0.0013	0.017	0.473	0.0013	0.016	0.240	0.009
GENIE3	0.608	0.0052	0.273	0.616	0.0046	0.256	0.608	0.0052	0.277	0.610	0.0055	0.275	0.591	0.0040	0.235	0.306	0.009
Global Silencing	0.605	0.0033	0.195	0.596	0.0037	0.208	0.640	0.0053	0.256	0.613	0.0040	0.217	0.608	0.0033	0.209	0.308	0.016
Network Deconvolution	0.624	0.0040	0.246	0.610	0.0046	0.254	0.624	0.0047	0.263	0.624	0.0045	0.257	0.659	0.0071	0.309	0.316	0.018
Proposed approach	**0.642**	**0.0053**	**0.276**	**0.643**	**0.0058**	**0.295**	**0.654**	**0.0055**	**0.284**	**0.644**	**0.0056**	**0.280**	**0.644**	**0.0043**	**0.252**	**0.325**	**0.004**

The first five sections contain the values for the AUROCs, AUPRs and the true positive rates (TPR) at relatively low value of false positive rate (FPR = 0.03) obtained based on the four different data sets and their combination by using the compared methods. The last two columns include the values for the OverallScore and the standard deviation of the obtained AUROCs. The ROC statistics are calculated using the R package minet. The entries in bold represent the best performer method with respect to the corresponding data set.

**Table 2:** Selector value - *E. coli* data sets.

Method	Cold	Heat	Oxidative	Lactose diauxie	All data sets
GGM	0.66	0.50	0.61	0.43	0.17
ARACNE	1	1	1	1	0.77
CLR	0.15	0.24	0.39	0.35	0.33
*L*_1_	0.70	0.70	0.68	0.69	0.67
*L*_1/2_	0.76	0.76	0.68	0.62	0.91
*L*_0_	0.78	0.76	0.70	0.62	0.90
GENIE3	0.65	0.63	0.93	0.79	0.94
Global silencing	0.31	0.27	0.69	0.43	0.36
Network deconvolution	0.17	0.35	0.27	0.23	0.45
Proposed approach	0.44	0.96	0.65	0.69	0.96

The columns contain the selector values for the networks reconstructed based on the four data sets and their combination by using the compared methods.

**Table 3:** Prediction of regulatory types - *E. coli* data sets.

Method	Cold				Heat				Oxidative				Lactose diauxie			
GGM	+	−	TN	TP	+	−	TN	TP	+	−	TN	TP	+	−	TN	TP
	0.027	0.016	0.994	0.006	0.029	0.019	0.994	0.002	0.029	0.017	0.995	0.004	0.026	0.022	0.995	0.004
ARACNE	—	—	0.937	0.088	—	—	0.963	0.102	—	—	0.969	0.063	—	—	0.887	0.242
CLR	—	—	0.968	0.028	—	—	0.968	0.039	—	—	0.968	0.033	—	—	0.968	0.027
*L*_1_	0.004	0	0.994	0.005	0.004	0	0.994	0.005	0.003	0	0.994	0.004	0.005	0	0.994	0.007
*L*_1/2_	0.003	0	0.994	0.005	0.003	0	0.994	0.005	0.003	0	0.994	0.004	0.007	0	0.994	0.006
*L*_0_	0.003	0	0.994	0.005	0.003	0	0.994	0.005	0.003	0	0.994	0.004	0.004	0	0.994	0.006
GENIE3	—	—	0.968	0.273	—	—	0.968	0.295	—	—	0.968	0.277	—	—	0.968	0.275
Global silencing	0.347	0.327	0.968	0.195	0.367	0.312	0.968	0.208	0.362	0.278	0.968	0.256	0.340	0.318	0.968	0.217
Network deconvolution	0.374	0.298	0.968	0.275	0.406	0.279	0.968	0.254	0.391	0.278	0.968	0.284	0.344	0.315	0.968	0.280
Proposed approach	0.247	0.156	0.968	0.246	0.247	0.156	0.968	0.256	0.247	0.156	0.968	0.264	0.247	0.156	0.968	0.257

The fraction of correctly predicted activating (+) and repressing (−) regulatory relationships with respect to the experimentally verified regulations from RegulonDB are presented for the four different data sets. The fractions of true positive (TP) and true negative (TN) edges irrespective of the regulatory type are also included. The ‘—’ is considered where the method is not able to infer the regulatory type
