# Supplementary material for: Gene regulatory network inference using fused LASSO on multiple data sets
Source: Sci Rep. 2016 Feb 11;6:20533. doi: 10.1038/srep20533 (PMC4750075; doi:10.1038/srep20533)
Supplement: Supplementary Information [file srep20533-s1.pdf]

Supplementary File:
Gene regulatory network inference using fused LASSO on multiple data sets

Nooshin Omranian[1,2], Jeanne M. O. Eloundou-Mbebi[1], Bernd Mueller-Roeber[2], Zoran Nikoloski[1,*]

[1] Systems Biology and Mathematical Modelling Group, Max Planck Institute for Molecular Plant Physiology, Am Muehlenberg 1, 14476 Potsdam, Germany

[2] Department of Molecular Biology, University of Potsdam, Karl-Liebknecht-Str. 24-25, Haus 20, 14476 Potsdam, Germany

*** E-mail: nikoloski@mpimp-golm.mpg.de**

Supplementary Figures

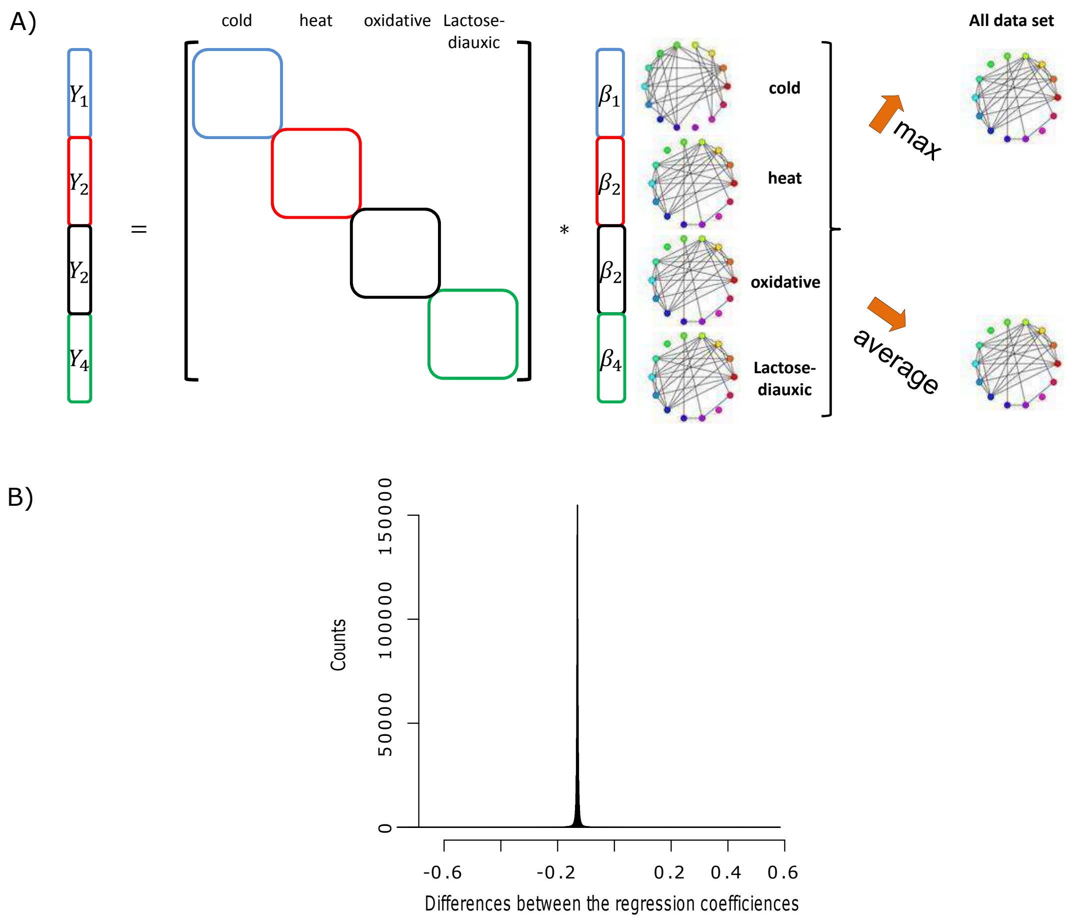

Figure S1. Inferred Network from all data sets by applying proposed approach (*E. coli* data sets). A) Applying the proposed approach on 'heat', 'cold', 'oxidative' and 'lactose-diauxic' data sets results in inferrence of four similar networks for each data set (in concordance with the fusion term in the proposed approach). Therefore, the gene regulatory networks from all data sets can be inferred either by apply maximum or average of the obtained edge weights from each data set. B) Histogram of the differences between the regression coefficients obtained based on each of the four data sets for the same response and regressor.

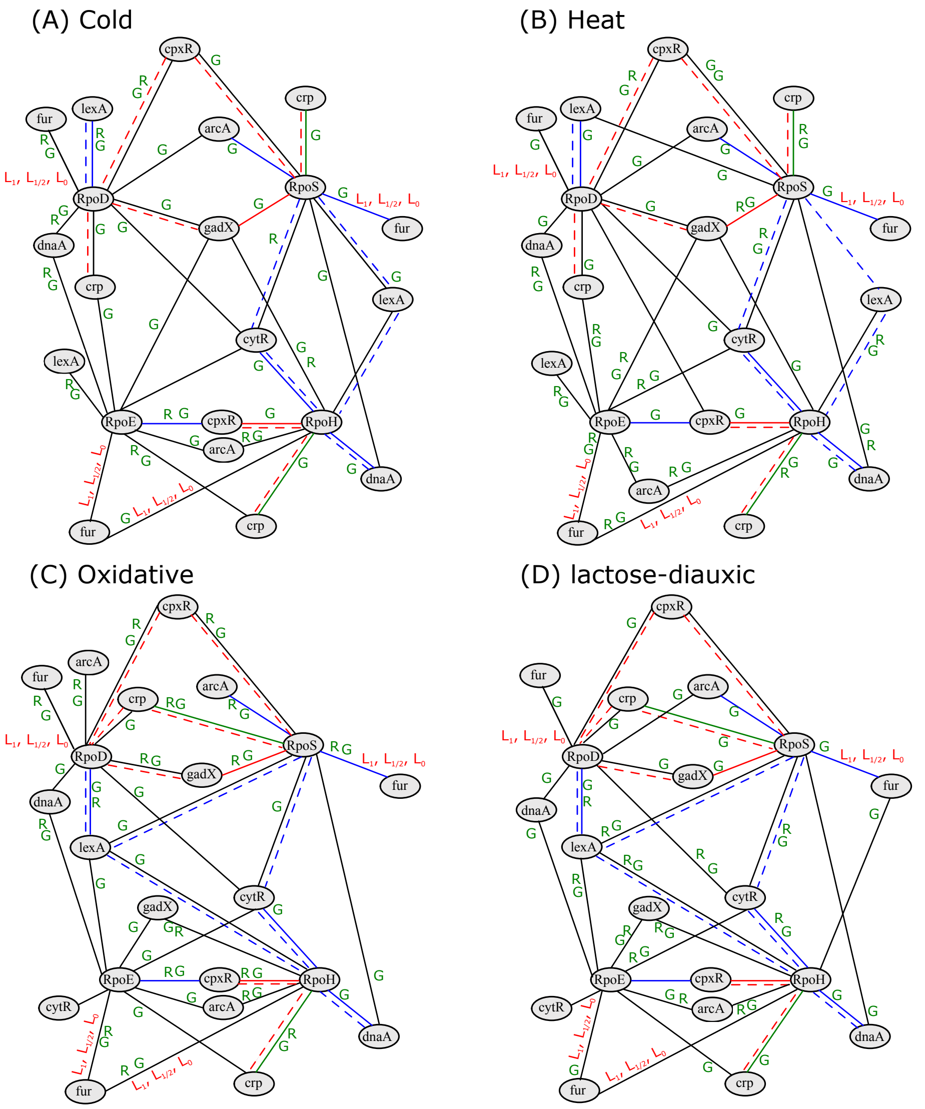

Figure S2. Sub-networks including sigma factors (*E. coli* data sets). The gene regulatory network for four sigma factors, RopD, RopE, RopH, and RopS, together with their experimentally verified interactions obtained from RegulonDB [1]. The colored edges belong to the subnetwork retrieved from RegulonDB, where red edges denote activating, while blue edges indicate repressing regulatory relationships. The edges marked in green are of unspecified regulatory type. If an edge was predicted by a method but is not included in the network from RegulonDB, it is colored in black. The predicted edges for L_1, $L_{1/2}$, and L_0 regularization-based models, CLR, and GENIE3 are marked by 'L_1', '$L_{1/2}$', 'L_0', 'R' and 'G', respectively, next to the corresponding edges. The letters are color-coded — red, blue or green fonts represent activating, repressing or unspecified relationships, respectively. The dotted edges denote the relationships predicted by the proposed approach. Illustrated are the predicted regulatory relationships and their types based on data from (A) cold, (B) heat, (C) oxidative stress, and (D) lactose-diauxic shift time-series experiments.

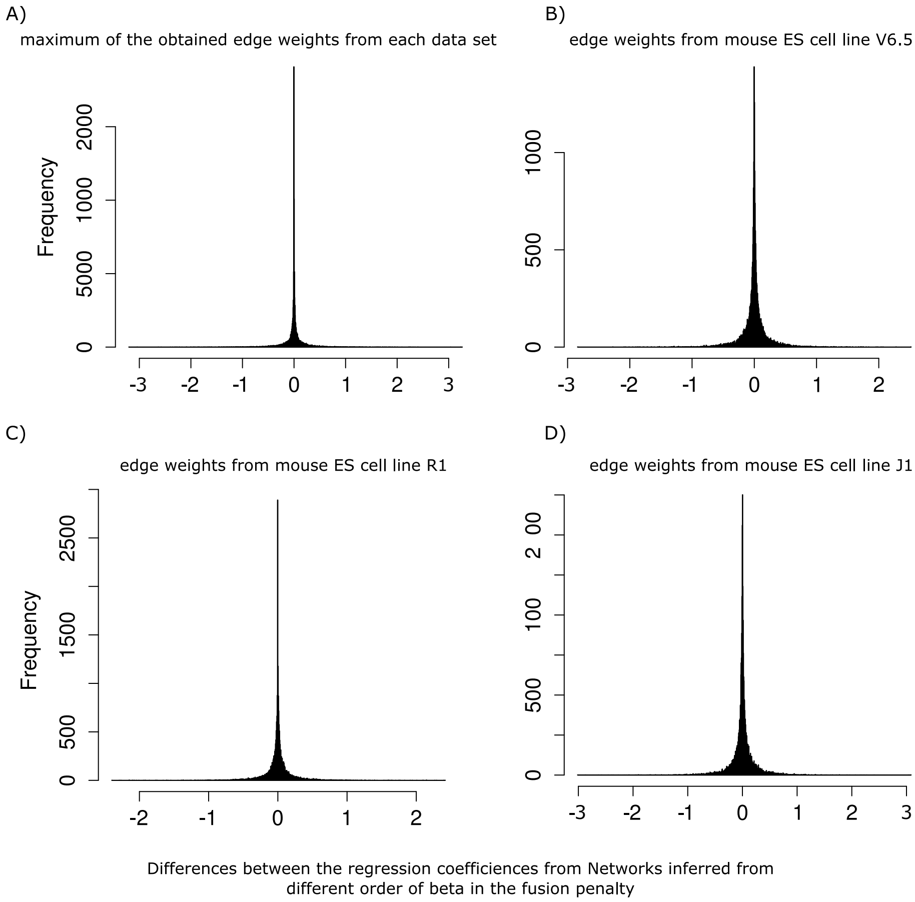

Figure S3. Histogram of the differences between the regression coefficients (*Mus musculus* data sets). The differences were calculated between the regression coefficients of the same regressor with all possible permutations (i.e., orderings) of the data sets. (A)-(D) Differences between the regression coefficients in networks inferred from all data sets, and each single data set from ES cell lines V6.5, R1, and J1, considering all possible orderings of data sets, respectively.

Supplementary Tables

Data set	Cold					Heat				
**Methods**	**AUROC**	**CI**	**Pvalue**	**padj**	**D**	**AUROC**	**CI**	**Pvalue**	**padj**	**D**
**GGM**	0.501	(0.500-0.502)	2.20E-16	4.4E-16	-33.0014	0.499	(0.498-0.500)	2.20E-16	4.4E-16	-34.8762
**ARACNE**	0.492	(0.489-0.495)	2.20E-16	4.4E-16	-32.6115	0.509	(0.505-0.512)	2.20E-16	4.4E-16	-28.6859
**CLR**	0.499	(0.496-0.501)	2.20E-16	4.4E-16	-31.8314	0.504	(0.501-0.507)	2.20E-16	4.4E-16	-30.6258,
L_1	0.500	(0.499-0.501)	2.20E-16	4.4E-16	-34.5666	0.500	(0.499-0.501)	2.20E-16	4.4E-16	-34.5666
$L_1/2$	0.500	(0.499-0.501)	2.20E-16	4.4E-16	-34.6178	0.500	(0.499-0.501)	2.20E-16	4.4E-16	-34.6178
L_0	0.500	(0.499-0.501)	2.20E-16	4.4E-16	-34.6178	0.500	(0.499-0.501)	2.20E-16	4.4E-16	-34.6178
**GENIE3**	0.621	(0.615-0.628)	2.51E-03	0.003273913	3.0224	0.632	(0.626-0.639)	1.84E-05	2.62857E-05	4.284
**Global Silencing**	0.582	(0.576-0.588)	6.41E-09	1.13118E-08	-5.8056	0.589	(0.583-0.595)	8.84E-08	1.47333E-07	-5.3492
**Network Deconvolution**	0.623	(0.616-0.629)	8.74E-04	0.001191818	3.3283	0.612	(0.606-0.618)	0.9274	0.9274	-0.0911
**Proposed approach** (taking the maximum of corresponding regression coefficients to obtain netwrork over all data sets)	0.607	(0.601-0.614)				0.612	(0.610-620)			
**Proposed approach** (taking the average of corresponding regression coefficients to obtain netwrork over all data sets)	0.607	(0.601-0.614)				0.612	(0.610-620)			

Data set	Oxidative					Lactose-diauxic				
**Methods**	**AUROC**	**CI**	**Pvalue**	**padj**	**D**	**AUROC**	**CI**	**Pvalue**	**padj**	**D**
**GGM**	0.502	(0.499-0.504)	2.20E-16	4.4E-16	-35.319	0.500	(0.499-0.501)	2.20E-16	4.4E-16	-34.6201
**ARACNE**	0.5	(0.499-0.501)	2.20E-16	4.4E-16	-32.7209	0.506	(0.501-0.511)	2.20E-16	4.4E-16	-26.5533
**CLR**	0.501	(0.498-0.504)	2.20E-16	4.4E-16	-32.8548	0.498	(0.496-0.501)	2.20E-16	4.4E-16	-33.3201
L_1	0.500	(0.499-0.501)	2.20E-16	4.4E-16	-34.7259	0.501	(0.500-0.502)	2.20E-16	4.4E-16	-34.1491
$L_1/2$	0.499	(0.499-0.500)	2.20E-16	4.4E-16	-34.8305	0.501	(0.500-0.502)	2.20E-16	4.4E-16	-34.2506
L_0	0.499	(0.499-0.500)	2.20E-16	4.4E-16	-34.8305	0.501	(0.500-0.502)	2.20E-16	4.4E-16	-34.2504
**GENIE3**	0.624	(0.617-0.630)	1.31E-01	0.1455556	1.5109	0.622	(0.616-0.629)	4.44E-02	0.05123077	2.0105
**Global Silencing**	0.613	(0.607-0.619)	0.4447	0.4600345	-0.7642	0.593	(0.587-0.599)	6.78E-06	1.07053E-05	-4.5006
**Network Deconvolution**	0.627	(0.620-0.633)	2.87E-02	0.03444	2.1875	0.625	(0.618-0.631)	1.01E-02	0.012625	2.5723
**Proposed approach** (taking the maximum of corresponding regression coefficients to obtain netwrork over all data sets)	0.617	(0.610-0.623)				0.613	(0.607-0.620)			
**Proposed approach** (taking the average of corresponding regression coefficients to obtain netwrork over all data sets)	0.617	(0.610-0.623)				0.613	(0.607-0.620)			

Data set	All data sets					summary		
**Methods**	**AUROC**	**CI**	**Pvalue**	**padj**	**D**	**Overall scores**	**mean**	**sd**
**GGM**	0.500	(0.499-0.501)	2.20E-16	4.4E-16	-33.4435	0.500398961	0.5004	0.001140175
**ARACNE**	0.501	(0.500-0.502)	2.20E-16	4.4E-16	-33.1778	0.501566159	0.5016	0.006503845
**CLR**	0.508	(0.505-0.511)	2.20E-16	4.4E-16	-28.2413	0.501986887	0.5020	0.004062019
L_1	0.501	(0.500-0.502)	2.20E-16	4.13E-16	-33.9932	0.50039976	0.5004	0.000547723
$L_1/2$	0.500	(0.499-0.501)	2.20E-16	4.13E-16	-34.5123	0.4999996	0.5000	0.000707107
L_0	0.500	(0.499-0.501)	2.20E-16	4.13E-16	-34.6178	0.4999996	0.5000	0.000707107
**GENIE3**	0.602	(0.596-0.608)	0.1706	0.1827857	-1.3702	0.620120415	0.6202	0.011054411
**Global Silencing**	0.589	(0.583-0.595)	9.02E-06	0.00001353	-4.4394	0.593107954	0.5932	0.01175585
**Network Deconvolution**	0.639	(0.632-0.646)	4.15E-11	7.78125E-11	6.5986	0.625140415	0.6252	0.009654015
**Proposed approach** (taking the maximum of corresponding regression coefficients to obtain netwrork over all data sets)	0.61	(0.602-0.615)				0.611791047	0.6118	0.003701351
**Proposed approach** (taking the average of corresponding regression coefficients to obtain netwrork over all data sets)	0.611	(0.604-0.617)	0.6056	0.6257	0.5164	0.611991503	0.612	0.003605551

Table S1. ROC-based statistics for the compared methods(*E. coli* data set). This table includes AUROC values and the corresponding confidence intervals (CI), p-values from the comparison between proposed method and the compared method at each row, the corresponding adjusted p-values and the D-statistics which are obtained based on the four different data sets and their combination by using the compared methods. The overall AUROCscore, mean, and standard deviation of the AUROCs for each method is summarized in the last three columns, respectively. The ROC statistics are calculated using "pROC" package in R. In columns "padj" and "D", the entities highlighted in blue indicate the cases in which the proposed method significantly outperformed the other compared methods while the entities highlighted in yellow show the cases that the corresponding compared methos significantly outperformed the proposed method and the non-highlighted entities show the cases that the performance of the proposed method and the respective compared methods are comparable.

Methods	Time
**GGM**	8.5”
**ARACNE**	15’
**CLR**	6”
**Regularization-based models**	2’ 44”
**GENIE3**	4’
**Global Silencing**	22”
**Network Deconvolution**	47”
**Proposed approach per each regression/gene model** (without cross validation)	3”
**Proposed approach per each regression/gene model** (with cross validation)	9’ 75”

Table S2. Time complexity of the proposed method and the compared methods.

	Top 100 highly ranked edges				Top 31 highly ranked edges			
**Data**	**Expr. values**		**LFC**		**Expr. values**		**LFC**	
**Methods**	**AUROC**	**AUPR**	**AUROC**	**AUPR**	**AUROC**	**AUPR**	**AUROC**	**AUPR**
**GGM**	0.476	0.080	0.571	0.102	0.408	0.067	0.429	0.095
**ARACNE**	0.483	0.078	0.409	0.063	0.494	0.081	0.443	0.067
**CLR**	0.444	0.074	0.418	0.065	0.469	0.076	0.492	0.077
$L1$	0.468	0.070	0.470	0.071	0.437	0.065	0.444	0.076
$L1/2$	0.506	0.076	0.509	0.079	0.421	0.062	0.441	0.070
$L0$	0.517	0.077	0.472	0.074	0.421	0.062	0.460	0.081
**GENIE3**	0.466	0.069	0.479	0.075	0.479	0.069	0.492	0.080
**Global Silencing**	0.558	0.087	0.454	0.073	0.615	0.100	0.472	0.075
**Network Deconvolution**	0.392	0.069	0.434	0.063	0.438	0.079	0.448	0.071
**Proposed approach** (taking the maximum of corresponding regression coefficients to obtain network over all data sets)	**0.598**	**0.102**	**0.607**	**0.112**	**0.561**	**0.100**	**0.570**	**0.103**

Table S3. ROC-based statistics for the compared methods (MTB data set). The left (right) four columns contain the values for the AUROCs and AUPRs obtained from the sub-networks including the top 100 (31) highly ranked edges predicted by the compared methods. To infer the gene regulatory networks, all the compared methods applied on both: the gene expression levels (Expr. values) and the obtained log2-transformed gene expression fold changes between the control (time point 0) and the hypoxia and re-aeration time-series samples (LFC).

Data	Expr. values							LFC						
**Methods**	**Rv0081 $\longrightarrow$ Rv3597c (Lsr2)**	**Rv0081 $\longrightarrow$ Rv3416 (whiB3)**	**Rv3133c $\longleftrightarrow$ Rv2034 (DosR)**	**Number of predicted true interactions in the top 100 highly ranked edges according to the gold standard network which includes 31 edges**	**Median rank**	**AUROC**	**AUPR**	**Rv0081 $\longrightarrow$ Rv3597c (Lsr2)**	**Rv0081 $\longrightarrow$ Rv3416 (whiB3)**	**Rv3133c $\longleftrightarrow$ Rv2034 (DosR)**	**Number of predicted true interactions in the top 100 highly ranked edges according to the gold standard network which includes 31 edges**	**Median rank**	**AUROC**	**AUPR**
**GGM**	——	——	0.108 $\longrightarrow$ and $\longleftarrow$ ——	10 (ranks above 0.4)	0.369	0.498	0.083	——	——	0.162 $\longrightarrow$ and $\longleftarrow$ ——	13 (ranks above 0.5)	0.557	0.547	0.097
**ARACNE**	0.645	——	—— $\longrightarrow$ and $\longleftarrow$ ——	4 (ranks above 0.6)	0.902	0.485	0.780	1.000	——	—— $\longrightarrow$ and $\longleftarrow$ ——	2 (ranks above 1.0)	1.000	0.447	0.068
**CLR**	0.719	——	—— $\longrightarrow$ and $\longleftarrow$ ——	7 (ranks above 0.2)	0.096	0.446	0.075	1.000	——	0.035 $\longrightarrow$ and $\longleftarrow$ 0.034	3 (ranks above 0.9)	0.074	0.439	0.067
L_1	——	0.258	—— $\longrightarrow$ and $\longleftarrow$ 0.959	7 (ranks above 0.6)	0.375	0.456	0.069	——	-0.001	-0.357 $\longrightarrow$ and $\longleftarrow$ -1.000	8 (ranks above 0.3)	0.250	0.483	0.073
$L_{1/2}$	0.279	0.456	—— $\longrightarrow$ and $\longleftarrow$ 1.000	8 (ranks above 0.8)	0.772	0.503	0.075	——	0.012	-0.366 $\longrightarrow$ and $\longleftarrow$ -1.000	10 (ranks above 0.3)	0.417	0.489	0.075
L_0	0.0301	0.465	—— $\longrightarrow$ and $\longleftarrow$ 1.000	9 (ranks above 0.7)	0.789	0.497	0.075	——	——	—— $\longrightarrow$ and $\longleftarrow$ -1.000	8 (ranks above 0.3)	0.390	0.477	0.074
**GENIE3**	0.198	0.336	0.045 $\longrightarrow$ and $\longleftarrow$ 0.185	4 (ranks above 0.5)	0.192	0.259	0.066	0.915	0.696	0.521 $\longrightarrow$ and $\longleftarrow$ 0.609	8 (ranks above 0.6)	0.384	0.508	0.080
**Global Silencing**	0.289	-0.107	-0.009 $\longrightarrow$ and $\longleftarrow$ 0.150	8 (ranks above 0.5)	0.262	0.554	0.087	-0.352	-0.271	-1.000 $\longrightarrow$ and $\longleftarrow$ -0.079	7 (ranks above 0.6)	0.306	0.439	0.071
**Network Deconvolution**	-0.624	-0.149	-0.133 $\longrightarrow$ and $\longleftarrow$ -0.105	5 (ranks above 0.7)	0.252	0.419	0.073	-0.423	0.037	-0.414 $\longrightarrow$ and $\longleftarrow$ -0.794	6 (ranks above 0.6)	0.306	0.453	0.065
**Proposed approach** (taking the maximum of corresponding regression coefficients to obtain network over all data sets)	-0.195	-0.639	-1.000 $\longrightarrow$ and $\longleftarrow$ ——	12 (ranks above 0.6)	0.6075	0.598	0.102	**-0.248**	**-1.000**	**-1.000 $\longrightarrow$ and $\longleftarrow$ -1.000**	**13 (ranks above 0.6)**	**0.379**	**0.601**	**0.110**

Table S4. Inference of GRN based on the gene expression values vs. log2-transformed gene expression fold changes (MTB data set). The left part contains the values obtained from the networks inferred by applying the compared methods on the gene expression levels (Expr. values) and the values in the right part obtained from networks inferred by applying the compared methods on the log2-transformed gene expression fold changes between the control (time point 0) and the hypoxia and re-aeration time-series samples (LFC). In each part, the first 3 columns include the predicted edge weights for the interactions: Rv0081 $\longrightarrow$ Rv3597c (Lsr2), Rv0081 $\longrightarrow$ Rv3416 (whiB3), and Rv3133c $\longleftrightarrow$ Rv2034 (DosR). The value '——' indicates that the interaction is not predicted by the corresponding method. Fourth column indicates the number of interactions and the minimum edge rank in overlap between the gold standard network (with 31 edges) and the top 100 highly rankd edges predicted by the compared methods. The last three columns include the values for the AUROCs and AUPRs obtained from the sub-network with edges above the median ranks of the predicted interactions.

Table S5. Top 100 highly ranked edges predicted by the proposed approach (MTB data set). The proposed approach has been applied to the log2-transformed gene expression fold changes between the control (time point 0) and the hypoxia and re-aeration time-series samples. The rows in bold represents predicted true interactions with respect to the gold standard network which includes 31 edges (page 180, [2]).

From	To	Weight
Rv0081	Rv2034	1
**Rv0081**	**Rv3416**	**-1**
Rv0324	Rv2021c	-1
Rv0324	Rv2034	1
Rv0494	Rv2989	-1
Rv0494	Rv3133c	1
Rv0735	Rv2989	1
Rv0735	Rv3133c	-1
Rv0757	Rv2703	-1
**Rv0757**	**Rv3133c**	**1**
Rv1221	Rv0324	1
Rv1221	Rv2989	-1
Rv1990c	Rv3133c	1
Rv1990c	Rv3416	-1
Rv2021c	Rv2989	-1
Rv2021c	Rv3416	1
**Rv2034**	**Rv2989**	**1**
**Rv2034**	**Rv3133c**	**-1**
Rv2324	Rv2021c	1
Rv2324	Rv3133c	-1
Rv2703	Rv0081	-1
Rv2703	Rv3286c	1
Rv2710	Rv2324	1
Rv2710	Rv3133c	-1

Rv2745c	Rv2989	1
Rv2745c	Rv3133c	-1
Rv2989	Rv2021c	-1
Rv2989	Rv3286c	1
**Rv3133c**	**Rv2034**	**-1**
Rv3133c	Rv3286c	1
Rv3249c	Rv2989	-1
Rv3249c	Rv3133c	1
Rv3286c	Rv0081	1
Rv3286c	Rv3416	-1
Rv3416	Rv2021c	1
Rv3416	Rv2989	-1
Rv3574	Rv2021c	-1
Rv3574	Rv2034	1
Rv3597c	Rv0081	-1
Rv3597c	Rv2703	1
Rv1221	Rv2021c	0.995548
Rv0757	Rv2324	0.99212
Rv0494	Rv3416	-0.9849
Rv2745c	Rv2034	0.98228
**Rv0757**	**Rv3416**	**0.95926**
Rv1221	Rv2324	0.946663
Rv3286c	Rv3133c	0.94583
Rv1221	Rv3249c	0.936648
Rv2745c	Rv2324	0.934762
Rv1990c	Rv0081	-0.92084
**Rv3133c**	**Rv0081**	**-0.9205**
Rv2710	Rv1221	0.920446
Rv0757	Rv2034	0.905974
Rv3133c	Rv1990c	0.873982

Rv1221	Rv2710	0.867457
Rv0757	Rv2021c	0.83551
Rv2710	Rv2021c	0.821087
Rv2703	Rv3133c	-0.80436
Rv3133c	Rv3249c	0.771523
Rv3249c	Rv3416	0.762466
Rv3416	Rv0081	-0.74848
Rv2710	Rv2034	0.746167
Rv2989	Rv3416	-0.72989
Rv3286c	Rv2324	0.700232
Rv3249c	Rv0081	-0.69548
Rv2989	Rv0081	0.686565
Rv2710	Rv2989	-0.67962
**Rv3597c**	**Rv3416**	**0.679355**
Rv3597c	Rv2021c	0.679267
Rv1221	Rv2703	0.668716
Rv0494	Rv0081	0.666786
Rv0494	Rv2703	-0.66321
Rv2710	Rv3416	0.662498
Rv0324	Rv0081	-0.65969
Rv3133c	Rv2324	-0.65965
Rv2324	Rv2034	0.654112
Rv1221	Rv1990c	0.652231
**Rv0757**	**Rv3597c**	**-0.63706**
Rv2021c	Rv2324	0.633763
**Rv3133c**	**Rv2021c**	**-0.63341**
Rv1221	Rv3133c	-0.63094
Rv3574	Rv0324	0.625078
**Rv0081**	**Rv0324**	**-0.62379**
**Rv0324**	**Rv3574**	**0.622689**

Rv2745c	Rv3416	0.62197
Rv0494	Rv0735	-0.60469
Rv0757	Rv2989	0.604439
Rv0757	Rv0324	0.588642
**Rv2989**	**Rv1990c**	**0.570885**
Rv2703	Rv3249c	0.565254
Rv2324	Rv2745c	0.550949
Rv3249c	Rv2021c	0.547925
Rv2710	Rv0324	0.545624
Rv2324	Rv1221	0.539971
Rv3574	Rv0735	-0.53539
Rv0324	Rv3133c	-0.53238
Rv3286c	Rv1221	0.528797
Rv1221	Rv0081	-0.52159
Rv0757	Rv2745c	0.516588
Rv0757	Rv3574	0.511315

Table S6. Top 100 highly ranked edges predicted by the proposed approach (MTB data set). The proposed approach has been applied to the gene expression levels from combination of both conditions: hypoxia and re-aeration. The rows in bold represents predicted true interactions with respect to the gold standard network which includes 31 edges (page 180, [2]).

From	To	Weight
Rv0081	Rv2989	1
**Rv0081**	**Rv3249c**	**-1**
Rv0324	Rv2703	-1
Rv0324	Rv3249c	1
Rv0494	Rv1221	1
Rv0494	Rv3286c	-1

Rv0735	Rv0324	-1
Rv0735	Rv2324	1
Rv0757	Rv0324	1
Rv0757	Rv2703	-1
Rv1221	Rv0757	-1
Rv1221	Rv2324	1
Rv1990c	Rv3286c	1
Rv1990c	Rv3416	-1
**Rv2021c**	**Rv3286c**	**-1**
Rv2021c	Rv3416	1
Rv2034	Rv2324	1
**Rv2034**	**Rv2989**	**-1**
Rv2324	Rv1221	1
Rv2324	Rv3286c	-1
Rv2703	Rv3286c	1
Rv2703	Rv3416	-1
Rv2710	Rv1221	1
Rv2710	Rv2989	-1
Rv2745c	Rv2324	1
Rv2745c	Rv3286c	-1
Rv2989	Rv2021c	-1
Rv2989	Rv3286c	1
**Rv3133c**	**Rv2034**	**-1**
Rv3133c	Rv3597c	1
Rv3249c	Rv0324	1
Rv3249c	Rv2989	-1
Rv3286c	Rv1990c	1
Rv3286c	Rv3416	-1
Rv3416	Rv2021c	1
Rv3416	Rv3286c	-1

Rv3574	Rv0324	1
Rv3574	Rv2989	-1
Rv3597c	Rv2034	1
Rv3597c	Rv3286c	-1
**Rv0081**	**Rv0324**	**-0.98865**
Rv2710	Rv2324	0.983986
Rv2989	Rv3416	-0.98109
**Rv0081**	**Rv1221**	**-0.96332**
Rv3249c	Rv3286c	0.963221
Rv2324	Rv2021c	0.920414
Rv3133c	Rv0324	0.914576
Rv0081	Rv2034	-0.91308
Rv0081	Rv2324	-0.91126
Rv2324	Rv2034	0.908568
Rv1221	Rv2034	0.880526
Rv3133c	Rv1990c	0.875233
**Rv0081**	**Rv2021c**	**-0.87431**
Rv0735	Rv3249c	-0.86799
Rv2021c	Rv1990c	-0.86285
Rv3597c	Rv2324	0.85272
Rv3416	Rv1990c	-0.84934
Rv2034	Rv1221	0.836681
**Rv3574**	**Rv3249c**	**0.832338**
Rv2710	Rv2034	0.818964
Rv3597c	Rv2021c	0.817438
Rv2710	Rv2021c	0.813795
Rv1221	Rv2710	0.79752
Rv2324	Rv3416	0.796901
Rv1221	Rv1990c	0.788825
Rv3574	Rv2034	0.77915

Rv0735	Rv3286c	0.772539
Rv3133c	Rv3574	0.769582
Rv2324	Rv2710	0.754181
Rv2703	Rv1990c	0.750657
Rv0081	Rv2710	-0.74529
Rv2745c	Rv1221	0.74224
Rv0494	Rv2034	-0.73674
Rv0324	Rv3416	0.735451
**Rv2989**	**Rv1990c**	**0.724917**
Rv3574	Rv2703	-0.72253
Rv2021c	Rv2324	0.719081
Rv3597c	Rv1221	0.716832
Rv1221	Rv2021c	0.683031
Rv3133c	Rv2324	-0.66865
Rv3133c	Rv3286c	0.667092
Rv0757	Rv2324	0.665919
Rv0735	Rv3574	-0.6628
Rv2989	Rv0324	-0.65529
Rv3249c	Rv2034	0.646604
Rv1221	Rv3249c	0.64338
Rv2989	Rv3249c	-0.6422
**Rv0081**	**Rv3416**	**-0.63855**
Rv2034	Rv3416	0.627478
Rv0494	Rv1990c	0.627001
Rv1990c	Rv2021c	-0.62403
Rv3416	Rv0324	0.622849
Rv2710	Rv3249c	0.621911
Rv3133c	Rv3249c	0.615456
**Rv0757**	**Rv3416**	**0.613604**
Rv0324	Rv2034	0.60786

**Rv0081**	**Rv3574**	**-0.60131**
Rv3286c	Rv2021c	-0.59653
Rv3249c	Rv1221	0.593896
Rv3416	Rv2324	0.589045

	First 500 highly ranked links		First 248 highly ranked links						
**Methods**	**AUROC**	**AUPR**	**AUROC**	**AUPR**	**Selector value**	**Number of predicted true interactions in the top 248 highly ranked edges according to the gold standard network which includes 248 edges**	**Median rank**	**AUROC**	**AUPR**
**GGM**	0.611	0.013	**0.613**	0.013	0.971	3 (score above 0.8)	0.405	0.596	0.012
**ARACNE**	0.376	0.007	0.376	0.007	0	0 (score above 0.0)	—	—	—
**CLR**	0.572	0.012	0.577	0.012	0.793	4 (score above 0.4)	0.086	0.577	0.012
**L1**	0.549	0.013	0.526	0.012	1.000	6 (score above 0.7)	0.076	0.523	0.012
**L1/2**	0.518	0.012	0.525	0.012	1.000	7 (score above 0.6)	0.149	0.519	0.012
**L0**	0.517	0.012	0.521	0.012	1.000	6 (score above 1.0)	0.272	0.526	0.012
**GENIE3**	0.61	0.015	0.472	0.01	0.785	3 (score above 0.6)	0.141	0.591	0.014
**Global Silencing**	0.486	0.016	0.408	0.014	1.000	9 (score above 0.5)	0.101	0.497	0.017
**Network Deconvolution**	0.559	0.017	0.461	0.012	1.000	8 (score above 0.6)	0.276	0.606	0.017
Proposed approach (taking the maximum of corresponding regression coefficients to obtain netwrork over all data sets)	**0.645**	**0.028**	0.593	**0.026**	1.000	**10** (score above 0.5)	0.209	**0.710**	**0.031**

Table S7. ROC-based statistics for the compared methods (*Mus musculus* data set). The first four columns contain the values for the AUROCs and AUPRs obtained from the sub-networks including the top 500 and 248 highly ranked edges predicted by the compared methods. The fifth column includes selector values, while the sixth columns contains the number of interactions and the minimum edge rank in overlap between the gold standard network (with 248 edges) and the top 248 highly rankd edges predicted by the compared methods. The last three columns include median edge rank as well as the values of the AUROCs, and AUPRs obtained from the sub-network with edges above the median ranks of the predicted interactions.

	First 500 highly ranked links							First 248 highly ranked links	
**Different permutation of beta in fusion penalty**	**AUROC**	**AUPR**	**selector value**	**shared between gold standard net and top 248 highly ranked edges**	**median**	**AUROC**	**AUPR**	**AUROC**	**AUPR**
**V6.5, R1, and J1**	0.629	0.027	1	8 (score above 0.50)	0.204	0.708	0.029	0.471	0.020
**V6.5, J1, and R1**	0.700	0.320	1	10 (score above 0.50)	0.201	0.735	0.032	0.601	0.026
**J1, R1, and V6.5**	0.645	0.028	1	10 (score above 0.50)	0.209	0.710	0.031	0.593	0.026
**J1, V6.5, and R1**	0.727	0.033	1	11 (score above 0.45)	0.189	0.724	0.031	0.625	0.027
**R1, J1, and V6.5**	0.647	0.028	1	7 (score above 0.50)	0.199	0.704	0.029	0.542	0.023
**R1, V6.5, and J1**	0.625	0.027	1	8 (score above 0.45)	0.176	0.672	0.026	0.547	0.023

Table S8. ROC-based statistics for the proposed approach considering all possible orderings of data sets (*Mus musculus* data set). To investigate the effect of the order in which the fusion penalty is formulated (Eq. 2), the networks were inferred from all possible permutation (i.e., orderings) of data sets from ES cell lines V6.5, R1, and J1. ROC-based statistics is obtained for the inferred networks. The first and the last two columns contain the values for the AUROCs and AUPRs obtained from the sub-networks including the top 500 and 248 highly ranked edges, respectively. The third column includes selector values, while the fourth columns contains the number of interactions and the minimum edge rank in overlap between the gold standard network (with 248 edges) and the top 248 highly rankd edges. The fifth, sixth and sevenths columns include median edge rank as well as the values of the AUROCs, and AUPRs obtained from the sub-network with edges above the median ranks of the predicted interactions.

Table S9. Top 248 highly ranked edges predicted by the proposed approach (*Mus musculus* data set). The rows in bold represents predicted true interactions with respect to the gold standard network which includes 248 edges.

From	To	Weight
cobra1	id1	1
cobra1	utf1	-1
foxd3	icam1	-1
foxd3	otx2	1
gsk3b	gli1	1
gsk3b	otx2	-1
hdac2	nanog	1
hdac2	zfp57	-1
irs1	ctgf	1
irs1	gbx2	-1
jmjd1c	ctgf	-1
jmjd1c	otx2	1
kdm3a	nr0b1	1
kdm3a	otx2	-1

kdm4c	ctgf	-1
kdm4c	lefty1	1
lin28	ctgf	1
lin28	kdm4c	-1
lyar	ctgf	-1
lyar	lin28	1
med12	nr0b1	-1
med12	otx2	1
myc	gbx2	1
myc	otx2	-1
**nanog**	**ccrn4l**	**1**
nanog	ier3	-1
nr0b1	ctgf	-1
nr0b1	kdm3a	1
paf1	fgf5	-1
paf1	id1	1
pou5f1	ctgf	-1
**pou5f1**	**kdm4c**	**1**
rexo1	gbx2	1
rexo1	ier3	-1
ring1	id1	1
ring1	ier3	-1
sall1	ctgf	-1
sall1	lefty1	1
sox15	ctgf	-1
sox15	nanog	1
**sox2**	**ctgf**	**-1**
sox2	otx2	1
t	ier3	1
t	nr0b1	-1

tcl1	ier3	-1
tcl1	nr0b1	1
thap11	ctgf	1
thap11	lefty1	-1
tpo	ctgf	-1
tpo	lefty1	1
trim28	ier3	-1
trim28	otx2	1
sall1	otx2	0.998191
myc	nr0b1	0.994031
jmjd1c	nr0b1	0.993554
irs1	lefty1	-0.97886
trim28	ccrn4l	0.975386
hdac2	lin28	0.97364
tpo	otx2	0.965116
nanog	kdm4c	-0.95078
trim28	ctgf	-0.93338
hdac2	tcl1	-0.92131
cobra1	nanog	-0.90666
nanog	klf2	0.895337
t	fgf5	0.888914
tpo	lef1	0.885853
hdac2	lefty1	0.875165
cobra1	nr0b1	-0.87326
hdac2	nr0b1	-0.86655
lin28	med12	-0.86421
irs1	kdm3a	-0.86205
t	lefty1	-0.85185
sall1	arid3b	0.850312
sall1	pou5f1	0.843607

paf1	otx2	-0.83078
irs1	icam1	-0.82986
foxd3	gli1	-0.81958
nanog	ctgf	-0.81524
gsk3b	nanog	-0.81518
hdac2	id1	0.814802
lyar	lefty1	0.809497
trim28	arid3b	0.80814
trim28	id1	-0.80408
lyar	ccrn4l	0.801667
sox2	gli1	0.80079
kdm3a	fgf5	-0.79515
med12	gbx2	-0.78196
cobra1	pou5f1	-0.77994
foxd3	klf2	-0.77542
lyar	fgf5	0.77201
tcl1	fbxo15	0.771791
sox2	ier3	-0.77036
irs1	kdm4c	-0.76846
tcl1	lefty1	-0.76713
tcl1	mta1	-0.76056
sall1	lyar	0.759416
ring1	lin28	0.758724
rexo1	med12	0.757257
t	tcl1	-0.7538
myc	fgf5	-0.74986
sox2	nr0b1	0.748822
ring1	kdm4c	-0.74419
trim28	lefty1	0.744028
nanog	lef1	-0.74266

thap11	id1	0.724673
nanog	id1	0.724383
nanog	otx2	-0.72432
lyar	otx2	0.72419
trim28	utf1	0.723498
rexo1	ctgf	-0.72259
**pou5f1**	**kdm3a**	**0.721875**
**irs1**	**pou5f1**	**-0.72085**
sall1	gli1	0.714289
tpo	arid3b	0.710113
sall1	pml	0.707441
tcl1	nanog	0.705104
sox2	id1	-0.70078
foxd3	fgf5	0.69929
t	gli1	-0.69127
sall1	myc	0.688972
lyar	gli1	0.686947
gsk3b	kdm3a	0.684777
thap11	tcl1	0.683537
hdac2	ccrn4l	-0.6812
foxd3	bmp4	-0.68064
sall1	ier3	-0.67898
paf1	tcl1	-0.67461
cobra1	ier3	0.674344
cobra1	lyar	-0.67288
lyar	arid3b	0.671975
myc	zfp57	0.666172
foxd3	arid3b	-0.6659
**pou5f1**	**lefty1**	**0.665518**
ring1	ctgf	0.665321

paf1	ier3	0.664585
myc	gli1	0.660251
ring1	t	0.659911
kdm3a	ctgf	-0.65946
jmjd1c	kdm3a	0.659404
sall1	utf1	0.654376
nr0b1	myc	0.638038
paf1	myc	0.632582
sall1	lef1	0.630213
pou5f1	otx2	0.628913
cobra1	fgf5	-0.62687
paf1	lefty1	-0.62646
med12	kdm3a	-0.62583
jmjd1c	ccrn4l	0.62565
ring1	fgf5	0.621203
nr0b1	otx2	-0.62091
lin28	myc	-0.62034
kdm3a	lefty1	-0.61999
gsk3b	irs1	0.619227
gsk3b	fgf5	-0.6178
nr0b1	ier3	-0.61715
lyar	ier3	-0.61298
thap11	icam1	-0.612
sox15	nr0b1	0.61106
jmjd1c	id1	-0.61021
thap11	zfp57	0.608631
trim28	myc	0.605681
sox2	lin28	0.60417
sox15	klf2	0.593295
nr0b1	fgf5	-0.59183

jmjd1c	kdm4c	0.590292
trim28	pou5f1	0.589716
thap11	nr0b1	-0.58843
hdac2	kdm3a	-0.58788
trim28	nr0b1	0.587344
rexo1	lin28	-0.58516
trim28	fbxo15	0.583367
med12	gli1	-0.58022
tcl1	gsk3b	-0.57684
cobra1	ccrn4l	-0.57641
hdac2	arid3b	0.576103
thap11	gli1	-0.57561
kdm4c	pou5f1	0.567775
hdac2	ier3	0.565329
med12	arid3b	0.564785
trim28	hdac1	0.562611
trim28	lyar	0.56059
gsk3b	sox2	-0.55982
irs1	gli1	-0.55893
trim28	irs1	-0.55568
foxd3	ier3	-0.55557
thap11	pml	-0.55175
gsk3b	utf1	-0.55171
trim28	pml	0.548891
jmjd1c	ier3	-0.54809
hdac2	gbx2	-0.5457
nr0b1	zfp57	0.545311
kdm4c	ier3	0.544126
cobra1	foxd3	-0.54294
nanog	tcl1	0.538544

tcl1	bmp4	0.537406
trim28	lef1	0.536891
med12	ctgf	0.536768
rexo1	ccrn4l	0.53293
foxd3	ctgf	0.529802
foxd3	mta1	-0.52974
sox15	id1	-0.5291
tpo	gbx2	-0.52512
trim28	icam1	-0.52506
trim28	hrc	-0.52464
ring1	fbxo15	-0.52413
myc	med12	-0.5224
nr0b1	gli1	0.518703
**nanog**	**sox2**	**0.517932**
sall1	nr0b1	0.51686
**sox2**	**lefty1**	**0.515403**
nr0b1	gbx2	0.50836
lyar	mta1	0.508312
**pou5f1**	**nanog**	**0.504579**
trim28	nanog	0.504474
t	ctgf	0.502968
sox2	pml	0.502767
myc	ier3	-0.50118
lyar	bmp4	-0.49879
tpo	nr0b1	-0.49801
nr0b1	tcl1	0.49767
cobra1	ctgf	0.493338
t	cd9	-0.49256
trim28	klf2	0.490387
hdac2	t	-0.48958

foxd3	id1	-0.48953
t	hdac2	0.486242
trim28	kdm4c	0.485992
t	arid3b	0.485924
gsk3b	ctgf	-0.4836
sox2	ccrn4l	-0.48307
kdm3a	med12	-0.48119
t	myc	0.480861
trim28	lin28	0.478559
jmjd1c	med12	-0.47676
thap11	cd9	0.474546
lin28	lyar	0.471547
tcl1	otx2	0.471019
sox15	tcl1	0.470854
lyar	lef1	0.46763
tcl1	ctgf	0.462992
hdac2	sox15	-0.46255
sall1	fgf5	0.462293
lyar	sox2	0.459458
paf1	kdm3a	0.458762
lin28	nanog	0.458637
paf1	gbx2	0.45837
nr0b1	fbxo15	0.454305
**foxd3**	**pou5f1**	**0.454007**

References

1. Gama-Castro, S. *et al.* Regulondb version 7.0: transcriptional regulation of *Escherichia coli* k-12 integrated within genetic sensory response units (gensor units). *Nucleic Acids Research* **39**, D98–D105 doi:10.1093/nar/gkq1110 (2011).

2. Galagan, J. E. *et al.* The *Mycobacterium tuberculosis* regulatory network and hypoxia. *Nature* **499**, 178–183 doi:10.1038/nature12337 (2013).
